# Transcriptomic meta-analysis reveals unannotated long non-coding RNAs related to the immune response in sheep

**DOI:** 10.3389/fgene.2022.1067350

**Published:** 2022-11-22

**Authors:** Martin Bilbao-Arribas, Begoña M. Jugo

**Affiliations:** Department of Genetics, Physical Anthropology and Animal Physiology, Faculty of Science and Technology, University of the Basque Country UPV/EHU, Leioa, Spain

**Keywords:** sheep, genomics, lncRNAs, RNA-seq, immune system, transcriptomics

## Abstract

Long non-coding RNAs (lncRNAs) are involved in several biological processes, including the immune system response to pathogens and vaccines. The annotation and functional characterization of lncRNAs is more advanced in humans than in livestock species. Here, we take advantage of the increasing number of high-throughput functional experiments deposited in public databases in order to uniformly analyse, profile unannotated lncRNAs and integrate 422 ovine RNA-seq samples from the ovine immune system. We identified 12302 unannotated lncRNA genes with support from independent CAGE-seq and histone modification ChIP-seq assays. Unannotated lncRNAs showed low expression levels and sequence conservation across other mammal species. There were differences in expression levels depending on the genomic location-based lncRNA classification. Differential expression analyses between unstimulated and samples stimulated with pathogen infection or vaccination resulted in hundreds of lncRNAs with changed expression. Gene co-expression analyses revealed immune gene-enriched clusters associated with immune system activation and related to interferon signalling, antiviral response or endoplasmic reticulum stress. Besides, differential co-expression networks were constructed in order to find condition-specific relationships between coding genes and lncRNAs. Overall, using a diverse set of immune system samples and bioinformatic approaches we identify several ovine lncRNAs associated with the response to an external stimulus. These findings help in the improvement of the ovine lncRNA catalogue and provide sheep-specific evidence for the implication in the general immune response for several lncRNAs.

## Introduction

Long non-coding RNAs (lncRNAs) are a heterogeneous class of genes that transcribe transcripts longer than 200 nucleotides lacking protein-coding potential (Ulitsky and Bartel, 2013). They are consistently transcribed, show lower expression and have less exons compared to protein-coding genes (PCGs). They are also more enriched in the nucleus and vary in their epigenetic marks and splicing efficiency (Derrien et al., 2012; Quinn and Chang, 2016; Melé et al., 2017). They show spatiotemporal-specific expression and epigenetic regulation, which highlights the diverse processes in which they are involved (Amin et al., 2015; Ransohoff et al., 2018). The expression of most lncRNAs varies greatly between individuals (Derrien et al., 2012; Kornienko et al., 2016). In the immune system lncRNAs are expressed in a very cell-specific and dynamic way, even within lineages of the same cell types (Hu et al., 2013; Ranzani et al., 2015; Agirre et al., 2019) and this cell-type specificity seems to be conserved among species (Washietl et al., 2014). Thus, lncRNAs emerge as potential regulators of immune system cell function and gene expression regulation, which should be finely coordinated for the generation of a correct immune response to external stimuli such as pathogens or vaccines.

Next-generation sequencing has expanded the mammal transcriptome attributing to thousands of poorly understood non-protein-coding transcripts the largest share of genes. There are many lncRNAs that may be involved in immune processes, but most of them remain functionally uncharacterised, especially in non-model species. Some lncRNAs might simply be transcriptional noise, but several others appear to be functional (Ma et al., 2019; Ramilowski et al., 2020). LncRNAs do not have a single molecular mechanism. They can regulate gene expression through interactions with proteins, RNA or DNA and their functions can often be directed by their location, sequence or secondary structure (Marchese et al., 2017). Sometimes the act of transcription itself has a local functional output, regardless of sequence, which could explain their low sequence conservation (Engreitz et al., 2016; Marchese et al., 2017). For instance, *IFNG* gene expression is regulated by the gene locus of an antisense lncRNA, but not by its non-coding product (Petermann et al., 2019).

The lncRNA catalogues of livestock species remain under-annotated compared to the mouse or human annotations (Kosinska-Selbi et al., 2020; Lagarrigue et al., 2021). Publicly available gene annotations contain more than ten thousand mouse and human lncRNA genes, while the sheep annotation contains 2,229 lncRNA genes in Ensembl v.105 and 4,442 lncRNA genes in NCBI Release 104. There is limited genomic overlap between both sources, most likely reflecting the highly specific expression of lncRNAs and the incompleteness of the current annotations (Lagarrigue et al., 2021). The annotation and functional characterisation of livestock lncRNAs is essential, since most trait-associated variants in livestock lie within non-coding genome regions (Weikard et al., 2017). In sheep, lncRNAs have been profiled across a multi-tissue dataset (Bush et al., 2018), but there are few functional studies investigating their involvement in the immune response and those are difficult to compare due to differences in naming and data availability (Jin et al., 2018; Bilbao-Arribas et al., 2021; Chitneedi et al., 2021).

The exponential increase in RNA sequencing datasets in the last years offers a valuable opportunity for posing novel scientific questions or improving the statistical significance of the analyses in a cost-efficient manner (Sielemann et al., 2020). This is specially suitable for the profiling of lncRNAs, due to their highly specific expression (Lagarrigue et al., 2021) and for the profiling of the gene expression signatures of immune responses (Sparks et al., 2016). There is a great interest in gene expression meta-analysis methods (Sweeney et al., 2017; Toro-Domínguez et al., 2021), which have been successfully applied to profile the transcriptional signatures across respiratory viruses (Andres-Terre et al., 2015) or vaccines (Li et al., 2014) in human blood samples. In livestock, few RNA-seq studies have utilized meta-analysis procedures to date, none of them in sheep (Keel and Lindholm-Perry, 2022).

In this study, we take advantage of the increasing number of high-throughput functional experiments deposited in public databases in order to uniformly analyse, profile unannotated lncRNAs and integrate 422 publicly available ovine RNA-seq samples, histone modification chromatin immunoprecipitation sequencing (ChIP-seq) samples and Cap Analysis of Gene Expression sequencing (CAGE-seq) samples of blood cells, lymphoid organs and other immune cells. We expand the lncRNA catalogue in sheep and identify the common expression signature of protein coding genes and lncRNAs during the immune response, evidencing the potential role of hundreds of lncRNA genes in immune functions.

## Materials and methods

### Data collection

We selected 929 RNA-seq sequencing runs belonging to 15 BioProjects from NCBI Sequence Read Archive (SRA), which were merged into 422 samples, by the following inclusion criteria: Samples from an immune system tissue (blood, immune cells or lymphoid organs), at least five samples from a single BioProject, pair-end sequenced using an Illumina platform and genome mapping rate above 60%. Sample metadata such as tissue type, age, breed, sex, library type or experimental treatment was collected from NCBI databases and published articles. Due to metadata ambiguity, the strandness of the samples was assessed with Kallisto (Bray et al., 2016) prior to pipeline execution.

Most samples originated from functional experiments that studied the immune response to vaccines or vaccine components (PRJEB26387, PRJNA454435, PRJNA559411), helminth infections (PRJNA291172, PRJNA433706, PRJNA268183, PRJEB33476, PRJEB45790, PRJEB44063), bacterial infection (PRJEB15872) and pro-inflammatory gene upregulation (PRJNA631066). Other transcriptomic studies were not related to the immune response but were used to improve the novel lncRNA identification and as unstimulated controls (PRJNA528905, PRJNA485657, PRJNA362606). Besides, we added samples from the sheep expression atlas (PRJEB19199), including samples from bone marrow derived macrophages stimulated with lipopolysaccharide (LPS). All samples were dichotomized into two treatment-groups: samples from immune-stimulated animals and unstimulated or control samples.

### Transcriptome assembly and quantification

**FIGURE 1:**
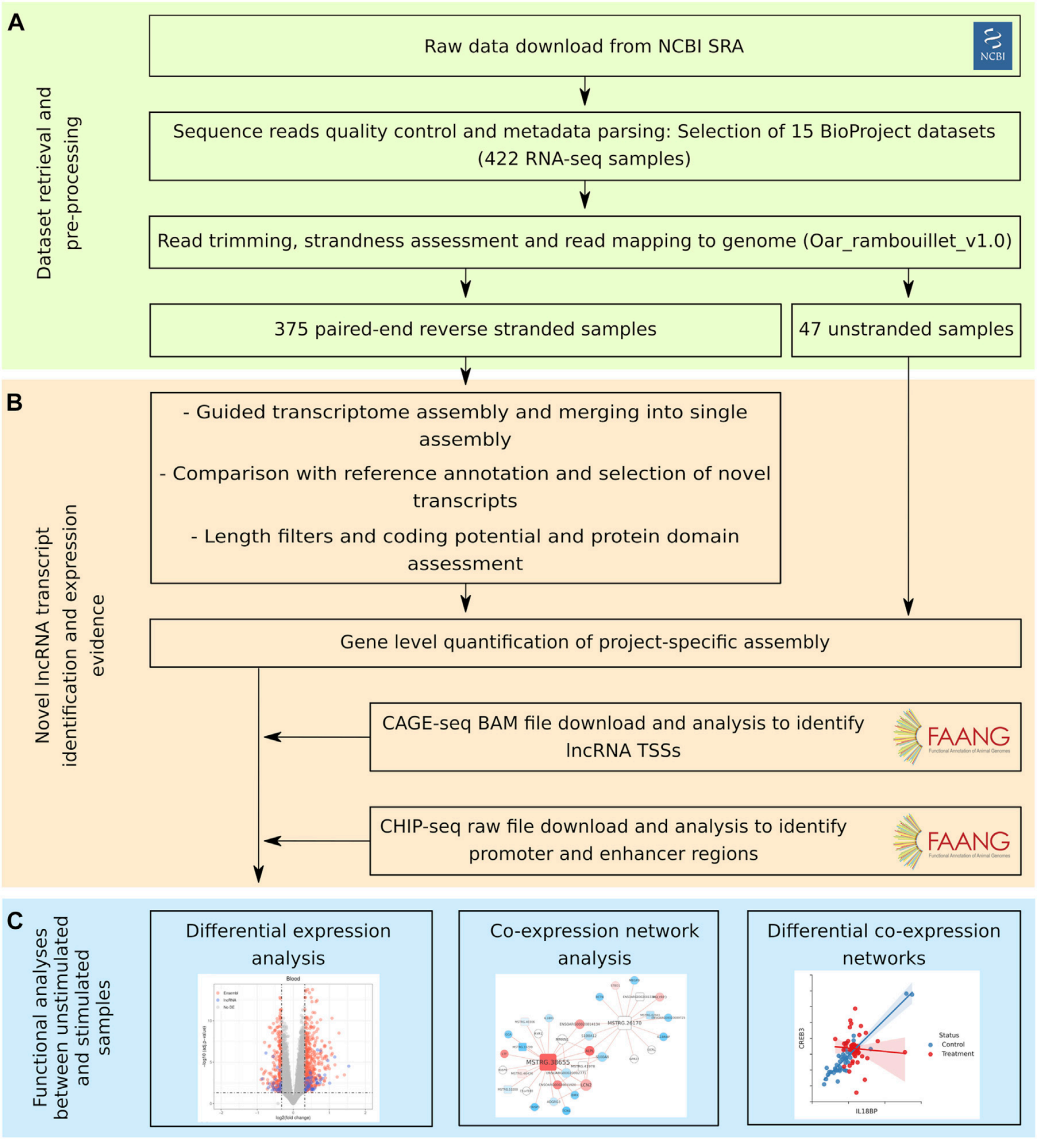
Bioinformatic workflow of the study. The workflow followed in this study can be divided into three sections. **(A)** First, sequencing data retrieval, preprocessing and mapping to the sheep genome. **(B)** Second, identification of unannotated lncRNA transcripts and evidence of expression. **(C)** Third, functional analyses between unstimulated samples and samples with an immune stimulation.

We downloaded and analysed the 422 RNA-seq samples with a uniform workflow using custom Snakemake v.6.15.1 (Köster et al., 2021) pipelines (Figure 1). 375 reverse stranded samples were used for transcriptome construction and novel lncRNA identification, while all samples were used for quantification based on the new transcriptome. Sequencing runs were downloaded from NCBI SRA with the SRA Toolkit and were merged into samples by their experiment ID. Adapter trimming and quality filtering was performed with cutadapt v.3.5 (Martin, 2011) using Illumina universal adapters and a phred threshold of 30. Reads were aligned to the sheep reference genome (Oar_rambouillet_v1.0) (Salavati et al., 2020) with STAR v.2.7.3a (Dobin et al., 2013) guided by the Ensembl (v102) annotation. StringTie2 v.2.0 (Kovaka et al., 2019) transcriptome assembler was used to reconstruct the transcriptome of each individual sample guided by the Ensembl (v102) annotation and with the--rf option. Then StringTie2 was applied again with the--merge option using all the transcriptomes in order to obtain a non-redundant transcriptome that is comparable between samples.

Quantification of gene expression was performed at transcript level with Kallisto v.0.48 (Bray et al., 2016) pseudoaligning the trimmed reads of all samples to the newly generated transcriptome, generated with GffRead v.0.11.7 (Pertea and Pertea, 2020). The--rf-stranded option was used with the 375 stranded samples.

### LncRNA identification

Potential novel lncRNAs were defined as unannotated transcripts that were located either in an intergenic region, in an intron of a known gene or in the antisense strand of a known gene. GffCompare v.0.11.2 (Pertea and Pertea, 2020) was used to compare the newly assembled transcriptome with the reference annotation and extract these transcripts. Single-exon transcripts longer than 500 nucleotides and shorter than 10kb, and multiexonic transcripts longer than 200 nucleotides and shorter than 50 kb were kept. The assessment of the coding potential of the candidate transcripts was done with three different tools. The coding potential prediction module of FEELnc (Wucher et al., 2017), based on a Random Forest classifier, was trained with sequences of bovine coding genes and lncRNAs from NONCODE database (Zhao et al., 2021). Coding-Potential Assessment Tool 3.0.2 (CPAT) (Wang et al., 2013) is a logistic regression-based tool that we trained and selected the classification threshold following authors’ instructions using the same bovine coding and non-coding sequences. HMMER 3.3.2 (Eddy, 2011) was used to detect Pfam protein domains in our potential lncRNAs, which were translated into the three possible frames. Transcripts classified as non-coding by FEELnc and CPAT and without protein domains detected by HMMER were kept. Transcripts classified by CuffCompare as a novel isoform of a known gene were also kept, as transcripts that had passed the coding potential tests could be legit non-coding isoforms. The selected transcripts were defined as the final set of novel lncRNAs.

Novel lncRNA transcripts were classified with a custom Python script (see Data Availability section) based on their position relative to their closest gene. Transcription start sites (TSSs) were defined as the start or stop nucleotides, depending on strandness. Seven classes were defined: 1) antisense, for those transcripts overlapping a gene in the opposite strand; 2) sense intronic or antisense intronic, for transcripts fully contained within an intron; 3) intergenic, for lncRNAs at least 5 kb away from any known gene; 4) divergent, with TSSs within 5 kb and in the opposite strand; 5) convergent, with transcription stops within 5 kb and in the opposite strand; 6) sense upstream, located less than 5 kb upstream of a gene and in the same strand; and 7) sense downstream, located less than 5 kb downstream of a gene and in the same strand.

To compare the novel lncRNAs with the recently upgraded ovine NCBI RefSeq annotation (release 104), which is based on the ARS-UI_Ramb_v2.0 new reference genome (Davenport et al., 2022), transcript coordinates were remapped with the NCBI Genome Remapping Service (https://www.ncbi.nlm.nih.gov/genome/tools/remap). They were compared with the NCBI lncRNAs using GffCompare (Pertea and Pertea, 2020). Transcripts models with codes “ = “, “j”, “c”, “k”, “o”,"m" or “n" were considered as overlapping, transcripts with codes “c” or “k” were considered compatible isoforms and transcripts with code “ = ” were considered exact matches.

### CAGE-seq and ChIP-seq data analysis

We downloaded the mapped BAM files of CAGE-seq experiments of five immune tissues from a multi-tissue project of sheep TSSs (tonsil, alveolar macrophages, spleen, mesenteric lymph node and prescapular lymph node) (Salavati et al., 2020) and analysed them using the same pipeline as the authors, with some modifications. In short, downloaded BAM files were converted to bigwig format with bedtools v.2.30.0 (Quinlan and Hall, 2010) and BedGraphToBigWig from UCSC tools (Kent et al., 2010). The R package CAGEfightR v.1.12.0 (Thodberg et al., 2019) was used for normalization and clustering of CAGE tags. CAGE tags <10 read counts were removed and all the remaining tags from any of the tissues were kept, to include tissue-specific TSSs. CAGEfightR was also used to identify bidirectional clusters. In order to get the genes supported by CAGE-predicted TSSs we used the BedTools python implementation pybedtools v.0.8.1 (Dale et al., 2011) to search for TSSs from the assembled transcriptome within 0.5 kb from them, accounting for strandness.

Sheep ChIP-seq sequencing files from alveolar macrophages (Massa et al., 2021) were downloaded from the NCBI Sequence Read Archive (SRA) and were analysed in an uniform way. Reads were aligned to the sheep genome (Oar_rambouillet_v1.0) with Bowtie2 v.2.3.5.1 (Langmead and Salzberg, 2012). SAM files were converted to BAM format with samtools v.1.7 (Danecek et al., 2021), and were sorted, filtered for quality and removed duplicate reads with sambamba v.0.6.6 (Tarasov et al., 2015). MACS2 v.2.2.6 (Zhang et al., 2008) was used to call narrow peaks for histone modifications with a FDR cut-off of 0.05 and consensus peaks from the pairs of animals were obtained with bedtools v.2.30.0 (Quinlan and Hall, 2010). In order to get the genes supported by ChIP-seq peaks we used pybedtools v.0.8.1 to search for TSSs from the assembled transcriptome within 0.5 kb from them.

### Conservation in terms of sequence

Sequence level conservation was performed with standalone BLASTn (BLAST v.2.9.0) (Camacho et al., 2009) by aligning the sheep lncRNA transcripts against the lncRNAs annotated in Ensembl Release 106 from five species: goat, cattle, pig, mouse and human. Because of the known low sequence conservation expected in lncRNAs, results were filtered by identity >50, query coverage >50, E-value > 1e-05 and it was required that the length differences between each pair of sequences was less than 50%. Visualization of the genomic context of conserved lncRNAs was performed with pyGenomeTracks 3.7 (Lopez-Delisle et al., 2021). The tracks for CAGE-seq data were constructed by merging all BAM alignment files with samtools (Danecek et al., 2021) into a single file and then was converted to bigwig format as previously. The tracks for histone modification ChIP-seq data were the consensus peaks obtained from MACS2.

### Analysis of gene expression

Kallisto abundance estimates were imported to R and summarized to gene level with IsoformSwitchAnalyzeR (Vitting-Seerup et al., 2019) in order to set confident gene identifiers for ambiguous transcripts. Counts of annotated genes and novel lncRNA genes were kept for further analysis, discarding potential novel unannotated coding genes. For gene expression data exploration, we normalized the estimated gene counts with the variance stabilizing transformation from DESeq2 (Love et al., 2014) and filtered out genes with less than 0.5 transcripts per million (TPM). The first two components of the principal component analysis (PCA) and the two first dimensions of the t-Distributed Stochastic Neighbor Embedding (t-SNE) were used for visualization. LncRNAs were tagged as expressed if they could be detected above 0.1 TPM or one TPM in at least 20% of the samples in a tissue group. Two-sided Mann-Whitney U tests were performed to compare expression means between classes.

Differential gene expression (DGE) was performed with DESeq2 (Love et al., 2014) using the estimated counts of annotated genes and lncRNA genes expressed in at least half of each sample groups and exported from IsoformSwitchAnalyzeR (Vitting-Seerup et al., 2019). Differential expression was tested separately in a blood and cell sample dataset on one side, and in a lymph node-only dataset on the other, because there were not stimulated samples from other lymphoid organs and that would unbalance the dataset. The Wald test was applied between unstimulated samples and stimulated samples using the effect of the interaction of tissue type and BioProject IDs as covariates for the lineal regression model, as those were the main drivers of the groupings seen in the exploratory analysis. Log_2_ fold change (log_2_FC) values from lowly expressed and highly variable genes were shrunken using the apeglm method (Zhu et al., 2019). Genes with an FDR-adjusted *p*-value lower than 0.05 and an absolute log_2_FC higher than 0.32, which corresponds to a 20% expression change, were kept. The relatively low log_2_FC filter was chosen because the large number of samples and the heterogeneity of the dataset produced differentially expressed genes with modest effect sizes and robust *p*-values.

Gene set enrichment analysis of differentially expressed genes was done with gProfiler R package (Raudvere et al., 2019). The statistical domain scope used was the list of all expressed genes for each tissue, in order to reduce the tissue type specific expression bias. Benjamini–Hochberg FDR correction was applied to the *p*-values and the threshold was set to 0.05.

### Co-expression analyses

Co-expression analyses were performed in the blood and immune cell dataset and in the lymph node-only dataset separately. Genes expressed in less than half of the samples were removed and strong outlier samples were removed in order to get a better fit to a scale-free topology. We tested two network construction pipelines: 1) The pipeline proposed by the authors of Gene Whole co-Expression Network Analysis (GWENA) (Lemoine et al., 2021), which consists of applying the variance stabilizing transformation (VST) from DESeq2 (Love et al., 2014) and using spearman correlations, and 2) counts adjusted with trimmed mean of M-values (TMM) factors followed by asinh transformation, Pearson correlations and network transformation by context likelihood of relatedness (CLR) (Johnson and Krishnan, 2022). Before creating the correlation matrices, normalised gene expression was corrected for covariates with limma’s removeBatchEffect function (Ritchie et al., 2015) to account for the effect of the interaction of tissue type and BioProject ID, as those were the main drivers of the groupings seen in the exploratory analysis. The 30% less variable genes were removed for network construction. Co-expression networks were constructed with GWENA (Lemoine et al., 2021) R package, which implements the Weighted Correlation Network Analysis (WGCNA) (Langfelder and Horvath, 2008) R package.

Modules of co-expressed genes were detected with the threshold power and clustering threshold calculated by GWENA and a minimum module size of 30. Modules were merged if their eigengene, the first principal component of the module, correlation was higher than 0.9. Modules were associated with overall immune stimulation or specific stimulation types by correlating their eigengene to those variables. To calculate the correlation *p*-value threshold, we generated 1,000 random gene modules ranging from 30 to 1,000 genes, correlated their eigengenes with the treatment variable and calculated the false positive rate (FPR). The *p*-value threshold with the FPR lower than 0.05 was 1e-02, but 1e-03 was chosen for more robustness. The genes in each module were tested for Gene Ontology (GO) term enrichment with gProfiler (Raudvere et al., 2019) R implementation, setting the statistical domain scope to all the genes in the co-expression network and a FDR-adjusted *p*-value threshold of 0.05.

The differential co-expression analysis was carried out by calculating the spearman correlations between all genes used in the co-expression network analysis separately in the unstimulated and the stimulated samples. The z-score method implemented in the dcanr v.1.12.0 R package (Bhuva et al., 2019) was used for testing the statistical differences between z-transformed correlation coefficients in both conditions. *p* values were adjusted for multiple hypothesis testing in order to select differentially correlated gene pairs. Differential co-expression networks (DCN) were visualized in Cytoscape v.3.8.2 (Shannon et al., 2003) by integrating the differential co-expression results, co-expression modules and differential expression results. For visualization, genes without gene names in the Ensembl annotation were named after their human orthologue according to Ensembl Compara.

## Results

### Dataset description

We collected and analysed 422 publicly available RNA-seq samples of tissues related to the immune system (Supplementary Table S1) using a uniform pipeline (Figure 1). In terms of immune response induction, 49.1% of the samples had been stimulated in some way. Blood samples, as whole blood or PBMCs, represented the 64.5% of the dataset, organs and lymph nodes the 30.3% and immune cell subsets the 5.2%. The mean age of the animals was of 1.32 years and 60.1% of the samples came from male sheep. There are 12 different breeds in the dataset, with three of them being crossbreed. Library selection is an important factor for lncRNA profiling because there are transcripts that are not polyadenylated. Around half of the samples were polyA-selected and half of the samples sequenced total RNA. Besides, samples had an average of 45 million reads, summing around 19 billion reads in total (Supplementary Figure S1). Unique genome mapping rate with STAR was of 84.6% on average and pseudoalignment rate to the new transcriptome with Kallisto was of 84.7% on average (Supplementary Figure S1). The assembled and merged transcriptome annotation contained 308750 transcripts, of which 41638 were from annotated transcripts and 36067 were from novel lncRNA transcripts identified by our pipeline. These transcripts correspond to 63364 genes, including 25472 annotated genes and 21223 novel genes with at least one lncRNA isoform.

**FIGURE 2:**
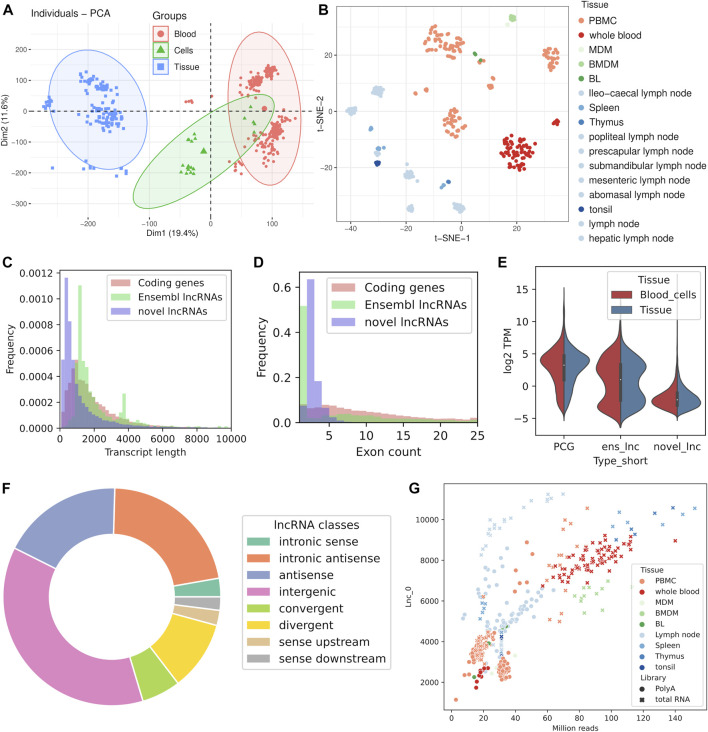
Characteristics of the dataset and the identification of lncRNAs. Exploratory analysis of all the samples included in the study using dimensionality reduction methods: **(A)** Principal Component Analysis (PCA) grouped by main tissue, **(B)** t-SNE plot with samples colored by tissue. **(C)** Transcript length distribution of PCGs and lncRNAs. **(D)** Exon length distribution of PCGs and lncRNAs. **(E)** Expression levels of PCGs and lncRNAs in blood cell samples and tissue samples. **(F)** Classification of lncRNAs into classes by genomic location. **(G)** Number of detected unannotated lncRNAs against sequencing depth.

All samples were clustered based on gene expression to assess the coherence of the data. Both clustering methods used clustered together the samples based on tissue, although intra-tissue groupings were influenced by the source project (Figures 2A,B). This could be expected as each study was performed in different conditions, with different breeds, ages, sex and protocols. Immune stimulation status did not affect much the clustering probably for the same reasons and because of the strong influence of tissue type.

### Novel lncRNA identification

We identified 21223 unannotated lncRNA genes from the sheep immune system samples that were assembled, and another 1724 annotated genes had novel non-coding isoforms classified as lncRNAs by our pipeline. Most of the novel genes with transcripts fulfilling the requisites to be classified as novel lncRNAs had all of their isoforms classified as such (17605). Some of the newly assembled gene models were coding genes missing from the Ensembl annotation that had non-coding isoforms, because they had novel transcripts with coding potential as well as lncRNA transcripts. Those unannotated genes and the 1724 annotated genes with novel non-coding isoforms were not considered as lncRNA genes for the gene-level expression analyses, even if individual transcripts could not be discarded as bona fide non-coding isoforms. We applied the same coding potential assessment methods used for novel transcripts to the annotated lncRNAs and discovered that there were transcripts potentially coding for a protein. This bias should be taken into account when comparing between the features of annotated and unannotated lncRNAs.

Regarding the characteristics of the novel lncRNAs, novel transcripts were shorter than the 2,229 lncRNAs annotated in Ensembl (Figure 2C) and a great proportion of them had two exons, in contrast to the Ensembl lncRNAs, which are monoexonic or have more than five exons (Figure 2D). We classified the novel genes based on position relative to known genes (Figure 2F, Supplementary Table S1). Intergenic lncRNAs (lincRNAs) were the most prevalent with 37% of the transcripts, followed by intronic antisense (22%) and antisense (18%) transcripts. Among the transcripts adjacent to annotated genes, the class of divergent lncRNAs was predominant (10% of all novel genes). The TSS of this kind of lncRNAs are very close to another gene’s TSS, which indicates that they probably arise from a single bidirectional promoter and may have implications in terms of gene expression regulation.

We explored the sequence-level evolutionary conservation of lncRNAs with other mammal species. Most lncRNAs are known to be poorly conserved in terms of sequence, but by detecting mammalian orthologues we provide further strength to the methods by which all unannotated lncRNAs have been identified. This analysis found a small number of conserved lncRNAs (Supplementary Figure S2; Supplementary Table S3). The biggest fractions of lncRNAs with conserved orthologues were found when comparing with goat and cattle lncRNA catalogues, with 11.9% and 7.3% of transcripts with significant hits, respectively. Comparing with the human and mouse catalogues, we found much less conserved lncRNAs. Interestingly, around 3% of novel lncRNAs, corresponding to 746 unique transcripts, matched with 392 unique human lncRNAs. Among these conserved lncRNAs, widely characterized lncRNAs such as *MALAT1*, *NEAT1*, *XIST*, *PACERR* or *FIRRE* were successfully detected in sheep (Supplementary Figure S3). Other conserved lncRNAs were those located in Hox gene loci, such as *HOTAIR*, *HOXA10-AS*, *HOXA-AS2* or *HAGLR*. Divergent lncRNAs were also among the conserved ones, like *FMNL1-DT*, *TOB1-AS1*, *EMSY-DT*, *RIPK2-DT*, *ATP8A1-DT* or *MAPK6-DT*. Despite not showing enough sequence similarity, we found some sheep transcripts located in the same divergent promoter as their human counterparts, for instance the putative orthologues of *HEATR6-DT* or *NIPBL-DT*.

Because of the recent improvement of the ovine NCBI reference genome and annotation (Davenport et al., 2022), the NCBI RefSeq lncRNA annotation was compared with the novel lncRNAs. After remapping to the new genome, out of the 4442 NCBI lncRNA genes, 1961 (44%) overlapped with an unannotated lncRNA. Exact matches of intron chains occurred in 571 transcripts, 238 transcripts were intron-compatible but differed in exon number and 3,679 where multi-exonic transcripts with at least one intron match. The overlap between Ensembl and NCBI lncRNAs was virtually inexistent. Thus, we detected around half of the annotated NCBI lncRNA genes using only immune-related tissues, even if most of the transcript models diverged in terms of splice-junctions.

### Expression patterns of lncRNAs

Expression levels of the novel lncRNAs detected in this study were lower than both protein coding genes and other annotated lncRNAs in the two main tissue categories (Figure 2E). In fact, after applying a minimum expression threshold in each tissue, expressed in at least 20% of the samples with one TPM, we were left with 2,267 expressed novel lncRNAs. Besides, we also detected 482 annotated lncRNAs above the expression threshold. Interestingly, 70% of the lncRNAs annotated by Ensembl were expressed in all three main tissue categories, while only 15% of novel lncRNAs were expressed in the three tissues (Supplementary Figure S4). Setting a less stringent mean expression threshold of 0.1 TPM results in 10045 expressed novel lncRNAs, 28% of them in all three tissues. Most of the novel lncRNAs (87%) and annotated lncRNAs (93%) could be detected in the set of lymphoid organs. Overall the overlap was greater between the blood samples and “immune cell” samples for both lncRNA genes and protein coding genes, as blood contains most of those cells (Supplementary Figure S4).

The amount of detected lncRNAs in each sample significantly correlated with sequencing depth for both unannotated lncRNAs (Pearson r = 0.75) and annotated lncRNAs (Pearson r = 0.85). Expression of PCGs was also correlated (Pearson r = 0.58) with sequencing depth but the saturation curve showed a flatter slope, meaning that it saturated earlier than lncRNAs (Supplementary Figure S5). The amount of lncRNAs expressed above 0.1 or one TPM got saturated above around 50 million reads, while the overall amount of expressed lncRNAs at any level did not saturate even at the highest sequencing depths in the dataset (above 100 million reads) (Figure 2G).

Divergent lncRNAs showed greater expression levels than other lncRNAs classes such as intergenic lncRNAs (Mann-Whitney U test *p*-value 2.9e-10) or antisense lncRNAs (Mann-Whitney U test *p*-value 1.3e-03), and only showed significantly lower levels than convergent lncRNAs (Mann-Whitney U test *p*-value 2.4e-03) (Supplementary Figure S6). Intronic antisense lncRNAs showed consistently lower expression than the rest of novel lncRNAs classes, in contrast with convergent lncRNAs, which were significantly more expressed than all other classes.

### Evidence of transcription by CAGE assays and histone modifications

Independent datasets of CAGE-seq and histone modification ChIP-seq were used in order to provide evidence of lncRNA transcription at RNA and DNA level. The CAGE-seq dataset contained samples from various lymphoid organs and alveolar macrophages, so it was used to provide support of expression in two sample subsets, blood and other immune cells, and lymphoid tissues. We obtained over two million significant CAGE peaks and around 30 thousand bidirectional CAGE peaks present in any of the five tissues.

**FIGURE 3:**
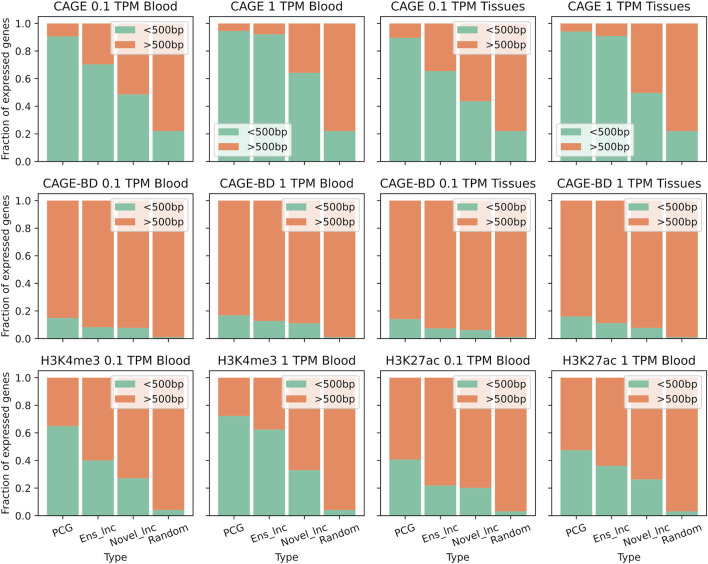
Support for transcription of annotated genes and novel lncRNAs. Fractions of expressed genes with detected TSSs or active gene histone modifications. TSSs were obtained from CAGE-seq peaks from five immune tissues and histone modifications were obtained from ChIP-seq peaks (H3K4me3 and H3K27ac) from alveolar macrophages. PCG: Protein coding gene, Ens_lnc: Ensembl lncRNA, Novel_lnc: Novel lncRNA.

In both sample subsets PCGs were more strongly associated with CAGE peaks than lncRNA genes, but reducing the analysis to the genes expressed above one TPM instead of 0.1 TPM increased the support in all gene types (Figure 3). This increase in support specially happened in lncRNAs. 64% and 50% of the TSSs of novel lncRNAs expressed above one TPM in the blood subset and the lymphoid subset, respectively, were located within500 bp of a CAGE peak. LncRNAs annotated by Ensembl reached a support level comparable to that of PCGs, with more than 90% of supported TSSs at one TPM. Bidirectional CAGE tag clusters are usually used to identify active enhancers because it is known that bidirectional transcription of short transcripts, known as enhancer RNAs (eRNAs), is a hallmark of enhancer activation (Andersson et al., 2014). Considering the genes expressed above one TPM or 0.1 TPM, novel lncRNAs were slightly less enriched in bidirectional clusters than PCGs. Around 11% and 8% of novel lncRNAs in the blood and tissue datasets, respectively, were transcribed from bidirectional sites (Figure 3). Some of them could be enhancer associated non-coding transcripts while others are divergent lncRNAs.

As for the ChIP-seq data, we analysed two histone modifications that are relevant for lncRNA transcription from a published dataset: H3K4me3, associated with promoters (Santos-Rosa et al., 2002), and H3K27ac, associated with active enhancers and promoters (Creyghton et al., 2010). The trend of H3K4me3 peaks from alveolar macrophages and CAGE peaks were similar regarding the genes expressed in blood and other immune cells, but the overlap between histone ChIP-seq data with TSSs randomly located in the genome was much lower (Figure 3). PCGs had the highest proportion of these promoter-associated marks followed by annotated lncRNAs and novel lncRNAs. Nevertheless, regarding the H3K27ac modification, the difference between lncRNAs and PCGs was smaller, which reflects the origin of many lncRNAs from enhancer-like regions. The support from this modification was similar in novel lncRNAs and annotated lncRNAs. 20% of the TSSs of novel lncRNAs expressed above 0.1 TPM in the blood subset were associated with H3K27ac. The apparent higher support for annotated non-coding models is probably linked with their misannotation.

Providing additional evidence of the transcription of novel transcripts assembled from short-read RNA-seq reads ensures that the detected genes are reproducible. We selected 12302 assembled gene models as bona fide lncRNA genes, those which were supported by at least one of the following: CAGE tags, histone modification ChIP-seq peaks or expressed above 0.1 TPM in at least 20% of the samples in a tissue group (Supplementary Figure S7). In this set, 47% of the lncRNAs had at least support from CAGE peaks or histone modifications. Around 1,000 lncRNAs were supported by all assays, including both histone modifications. The annotation files with all unannotated lncRNA transcripts, the set of high confidence transcripts and expression values can be found in a public repository (Bilbao-Arribas and Jugo, 2022).

### Differentially expressed lncRNAs and PCGs

**FIGURE 4:**
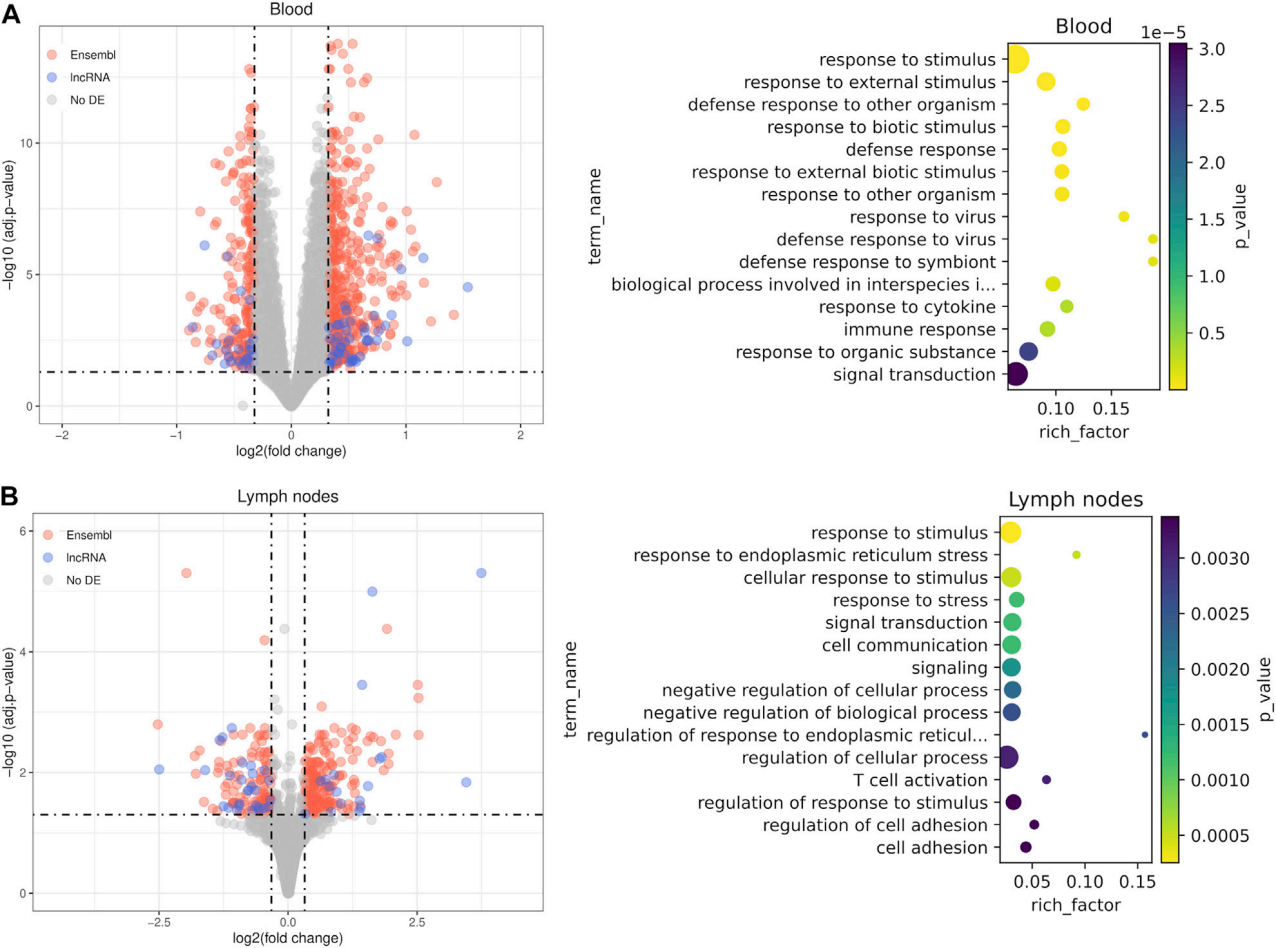
Differential expression results between stimulated samples and unstimulated samples. Analyses were performed in blood cell samples **(A)** and lymph node samples **(B)**. For each comparison, a volcano plot using shrunken fold changes and a dot plot with the results of gene ontology enrichment analysis (GO biological processes) are shown.

We performed differential expression analysis between unstimulated or control samples and samples stimulated with either vaccines or a pathogen in order to identify common lncRNAs induced during an immune response. In blood samples there were 716 differentially expressed genes, including 75 novel lncRNAs and 22 annotated lncRNAs (Figure 4A; Supplementary Table S4). The large number of samples used in the blood sample dataset 222) and the heterogeneity of the data produced many differentially expressed genes with modest effect sizes but robust *p*-values (Supplementary Figure S8). The most significant enriched terms among the known genes were biological processes related to the immune response to external pathogens such as *response to external stimulus* (GO:0009605, FDR = 2.86e-09), *response to virus* (GO:0009615, FDR = 6.75e-07) or *defense response* (GO:0006952, FDR = 1.19e-07). In lymph node samples, there were 365 differentially expressed genes, including 46 novel lncRNAs and 13 annotated lncRNAs (Figure 4B; Supplementary Table S4). In this case, among the most significant enriched terms with the highest quantity of genes were general terms such as r*esponse to stress* (GO:0006950, FDR = 2.51e-04) and *response to stimulus* (GO:0050896, FDR = 1.21e-03). More specifically, the terms related to T cell activation, like *T cell activation* (GO:0042110, FDR = 3.07e-03) and *regulation of T cell activation* (GO:0050863, FDR = 3.37e-03), reflect the critical roles of lymph nodes in adaptive immunity. Besides, there also were highly significant but smaller in size enriched terms related to response to endoplasmic reticulum (ER) stress.

There were 22 differentially expressed genes common to both datasets, among them an annotated lncRNA and an unannotated lncRNA. Some of the common PCGs are directly related with immunity, like *IL21*, which encodes a well known cytokine with immunoregulatory activity that induces proliferation and differentiation in several immune cell types. Other genes are related to apoptosis and inflammation (*MT2*, *IKBIP*, *AEN*, *OSGIN1*) and ER regulation (*WFS1*, *SELENOS*). Despite relatively similar number of DE genes in both comparisons, there is a big set of highly significant genes with effect sizes smaller than the threshold in the blood samples and many statistically significant but lowly expressed novel lncRNAs did not pass the fold change threshold because they were shrunken (Supplementary Figure S8). These results give support for potential involvement of a fraction of the detected novel and annotated lncRNAs in both the innate and adaptive immune responses, following the guilt-by-association principle.

### Co-expression network analyses detect immune-enriched gene signatures

**FIGURE 5:**
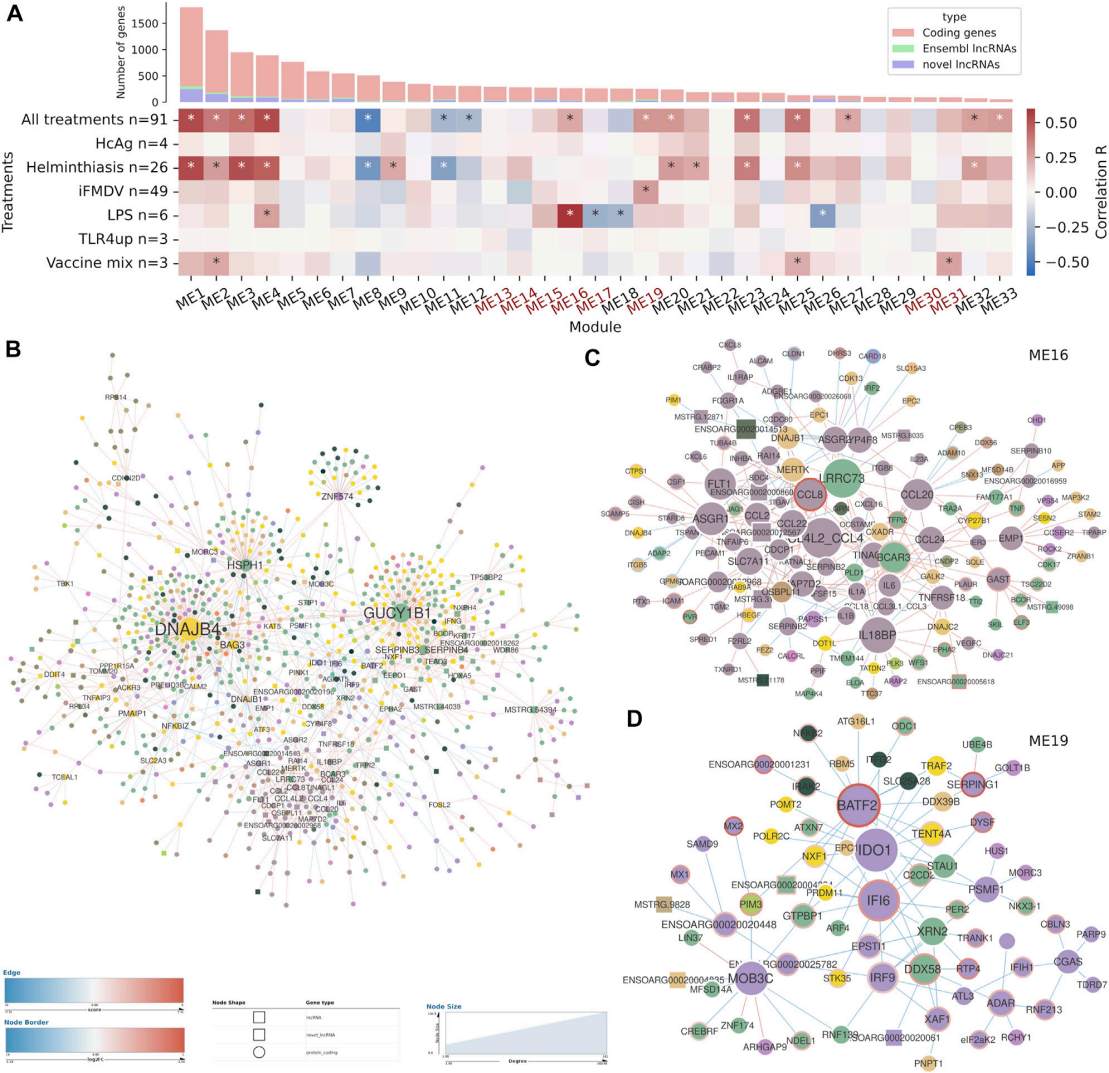
Co-expression analysis and differential co-expression network results in blood cell samples. **(A)** Correlations of gene co-expression modules with all stimulations and with each individual stimulation. Modules enriched in immune genes are highlighted in red. Number of genes in each module is depicted as a bar plot. **(B)** The full differential co-expression network. Node size is proportional to connectivity and differential associations are coloured by gain or loss of correlation strength. The edges of differentially expressed genes are coloured by fold change. **(C)** Sub-network with the differentially associated genes in module ME16. **(D)** Sub-network with the differentially associated genes in module ME19.

**FIGURE 6:**
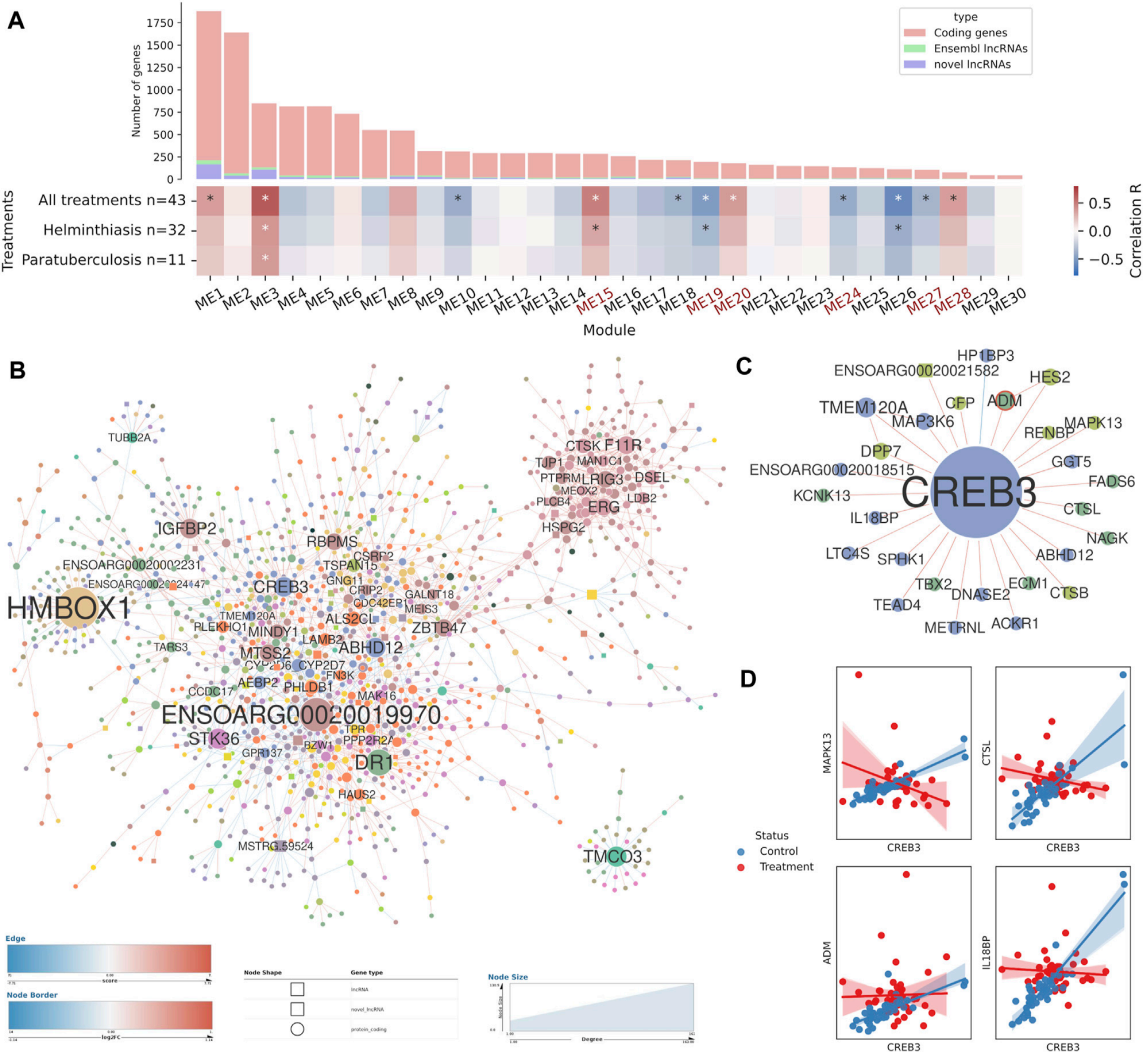
Co-expression analysis and differential co-expression network results in lymph node tissue samples. **(A)** Correlations of gene co-expression modules with all stimulations and with each individual stimulation. Modules enriched in immune genes are highlighted in red. Number of genes in each module is depicted as a bar plot. **(B)** The full differential co-expression network. Node size is proportional to connectivity and differential associations are coloured by gain or loss of correlation strength. The edges of differentially expressed genes are coloured by fold change. **(C)** The genes differentially co-expressed with *CREB3* transcription factor. **(D)** Individual examples of statistically significant differential associations between *CREB3* and four genes.

Gene co-expression networks were constructed providing valuable information about the expression relationships of lncRNAs with PCGs and allowing the inference of their putative functions by guilt-by-association. We tested two different network construction pipelines and selected the one proposed by the authors of GWENA (Lemoine et al., 2021), as it produced networks with better fit to a scale-free topology and most of the genes could be associated to an expression module. Covariate correction for tissue type and source project enabled the construction of unbiased networks (Supplementary Figure S9.10). Filtering of lowly expressed genes, genes with low variability and outlier samples that reduced the fit to a scale-free topology resulted in co-expression networks of 12898 and 13428 genes in blood samples and lymph nodes, respectively. In the blood dataset, genes with similar expression patterns were clustered in 33 modules ranging from 54 to 1832 genes (Figure 5A; Supplementary Table S5), and in the lymph node dataset genes were clustered in 30 modules ranging from 44 to 1909 genes (Figure 6A; Supplementary Table S5). Most modules included novel lncRNAs and annotated lncRNAs, and some of them were even hub genes of their module.

We searched for significant correlations among module eigengenes, the principal component of the genes in the module that depicts its dominant trend, and treatment variables. In the blood sample dataset, 15 modules were correlated (p-val < 1e-03) with the general treatment variable, which accounts for any kind of sample stimulation (Figure 5A). Considering correlations to specific immune stimulations, helminth infection shared many correlated modules with the general treatment variable, which meant that it was one of the main drivers of variability in the dataset. Stimulation with LPS and with inactivated foot-and-mouth disease virus (iFMDV) were correlated with specific gene modules different to those correlated to helminth infection. Other stimulations were also correlated to some modules but because of their small sample size they were not further taken into account.

Gene expression modules were characterised by GO term enrichment (Supplementary Table S5). Two of the stimulation-correlated modules (ME16 and ME19) were highly enriched in biological processes related to the immune response but they were not correlated with helminth infection. ME16 was associated with the sum of all treatments and was specially strongly correlated with LPS stimulation. The most significant enriched biological process GO terms were related to the general immune response, like *immune system process* (GO:0002376, FDR = 2.27e-07) or i*mmune response* (GO:0006955, FDR = 1.15e-05) and to cell migration and locomotion, including the terms *positive regulation of locomotion* (GO:0040017, FDR = 1.55e-06) and *leukocyte migration* (GO:0050900, FDR = 1.16e-05). ME19 was also associated with the sum of all treatments and was correlated to iFMDV treatment. It contained a high amount of immune response genes, for instance, from the 155 genes with GO annotations, 35 were related to *response to virus* (GO:0009615, FDR = 9.59e-25) and 56 to *immune system process* (GO:0002376, FDR = 1.56e-10). Besides, terms related to type I interferon response and signalling were also abundant.

In the lymph node network, the eigengenes of 11 modules were correlated (p-val < 1e-03) with the combined treatment variable (Figure 6A). The two available immune stimulation conditions, helminth infection and paratuberculosis, were correlated with a few modules, but several other significant modules emerged from the combined treatment variable correlation. The characterisation of gene expression modules by GO term enrichment revealed up to five immune-enriched modules: ME15, ME19, ME24, ME27 and ME28. Among them, the positively correlated modules showed functions involved in the innate immune response and general immune terms. For instance, in module ME15 the terms *immune response* (GO:0006955, FDR = 2.10e-11) or i*nnate immune response* (GO:0045087, FDR = 5.77e-07) are highly significant. In contrast, the negatively correlated modules are enriched in adaptive immune response terms. ME19 is enriched in GO terms related with T cell activation and lymphocyte proliferation while ME27 is enriched in terms related to B cell activation and proliferation. The lncRNAs present in the immune-enriched modules from both co-expression networks were classified as immune response-related lncRNAs.

In addition to the immune-enriched gene modules, another big module stood up (ME3), as it was correlated with both helminth infection and paratuberculosis. Most of the enriched GO terms were related to endoplasmic reticulum (ER) stress and protein post-translational processing, with terms like *response to endoplasmic reticulum stress* (GO:0034976, FDR = 2.60e-13), *Golgi vesicle transport* (GO:0048193, FDR = 2.42e-07) or *response to unfolded protein* (GO:0006986, FDR = 1.72e-06).

### Differential co-expression networks to identify regulatory relationships

Gene level differential co-expression, the gain or loss of correlation between two genes in different biological situations, indicates changes in regulatory relationships between those genes, which are often not evident from DGE results. All gene-pairs used in the co-expression network construction were tested for significant changes in correlation between control and stimulated samples and differential co-expression networks (DCN) were constructed with statistically significant gene-pairs (Supplementary Table S5). The DCN from the blood sample dataset contained 1,589 differential associations (FDR <0.05) among 1,348 genes (Figure 5B) and the DCN from lymph nodes contained 2,137 differential associations (FDR < 1e-03) among 1784 genes (Figure 6B). Both networks included around 60 lncRNAs each. In terms of network topology, networks showed a small amount of nodes (genes) with many edges (differential associations), while the rest of the nodes were more loosely connected to the network. Just around 5% of the nodes had 10 or more edges. Some very interconnected nodes formed clusters according to the gene co-expression modules from the previous analysis, but most of the topology was driven by a few high-degree nodes.

Specific differential associations were observed by individually inspecting each DCN. The blood sample network was centred on two high-degree genes that had more than 100 differential associations each but did not have obvious biological relationship with the immune response: *DNAJB4* and *GUCY1B1*. Among the rest of the 42 high-degree genes, defined as those with more than five differential associations, there were some lncRNAs and several immune genes such as *BATF2*, *IDO1*, *IFI6*, *IL18BP*, *NFKBIZ* and various CC chemokines. Focusing on the immune-enriched co-expression modules, many genes from the module ME16, most of them immune-related, formed a very interconnected subnetwork (Figure 5C). Even though the genes from this subnetwork were already correlated, they predominantly showed positive z-scores, which means that the correlations were stronger in the control samples than in stimulated samples, On the contrary, genes from the module ME19 did not form a separate cluster, but they showed negative z-scores, which means that their expression was correlated in the stimulated samples. Interestingly, many genes were up-regulated in the differential expression analysis. In the subnetwork composed by selecting the genes from this module and their differentially co-expressed pairs, there were transcription factor coding genes related to the immune response: *BATF2*, *IRF9* and *NFKB2* (Figure 5D). For instance, *BATF2*, upregulated in stimulated samples, is a transcription factor that controls the differentiation of lineage-specific cells in the immune system and immune-regulatory networks. There were several interferon-stimulated genes such as *IFI6*, *MX1*, *MX2*, *ADAR*, *EIF2AK2*, *IRF9* or *IFIH1*, all related to antiviral functions and upregulated in the stimulated samples.

The DCN obtained from lymph node samples did not contain many immune-related genes (Figure 6B). There were 187 high-degree genes, including 18 transcription factors coding genes that were potential drivers of the differential co-expressions, like *HMBOX1*, *CREB3*, *NFATC4*, *NFIB* or *EBF4*. *NFATC4*, for instance, is involved in T-cell activation, stimulating the transcription of *IL2* and *IL4* cytokine genes. *CREB3*, among many other functions, plays a role in the response to ER stress by promoting cell survival, a process that was previously found enriched in a co-expression module of which *CREB3* was not part of. *CREB3* was a high-degree node, differentially associated with 27 other genes (Figure 6C), and it showed mostly positive z-scores, thus, its expression was correlated in the unstimulated samples but those correlations were lost upon stimulation by helminth infection and paratuberculosis. Examples of differentially associated genes include *IL18BP*, *CTSL*, *MAPK13* and *ADM* (Figure 6D). *ADM*, which codes for a known lymphangiogenic factor, was upregulated in stimulated samples and its expression decoupled from that of *CREB3* in those samples. This DCN also contained several lncRNAs and 12 of them were high-degree nodes (7 known lncRNAs and 5 novel lncRNAs).

### Integration of evidence for lncRNA expression and function

We used differential gene expression analysis, co-expression analysis and differential co-expression network analysis for the functional association of lncRNA genes with the activation of the immune response. Those three approaches resulted in 320 lncRNAs associated in at least one analysis (Supplementary Figure S11). The differential expression between stimulated and unstimulated samples showed the highest number of immune response-associated lncRNAs. Interestingly, the histone modification support in differentially expressed novel lncRNAs was much higher than in the whole set of novel lncRNA genes, 49% against 19%, and the trend was similar in the case of CAGE support. A summary of all transcription evidence and associations in an analysis for each lncRNA is available as a supplementary file (Supplementary Table S6).

## Discussion

Using 422 RNA-seq samples from ovine immune tissues, we assembled a project-specific transcriptome and retrieved 17605 unannotated lncRNA loci. Around 70% of those novel genes were expressed in a sufficient number of samples and/or were supported by histone modifications or TSSs from independent experiments. LncRNAs are usually annotated with evidence-based methods, because they lack sequence features like conservation or complete ORFs (Uszczynska-Ratajczak et al., 2018), and this evidence mostly comes from mapping sequencing reads to the genome of interest. Model organisms have been annotated *via* manual curation of a variety of assays, but in the absence of this kind of data in livestock species, lncRNA annotations usually rely on automated short-read transcriptome assemblies. Second generation sequencing short-read RNA-seq is widely used because of its high yield and low cost (Mudge and Harrow, 2016) and has been used in many lncRNA annotations (Uszczynska-Ratajczak et al., 2018), but using this kind of data is challenging, because the nature of short reads makes it difficult to completely characterize the structure of non-coding transcripts (Conesa et al., 2016).

For higher confidence on the assembled transcripts, only paired-end samples were used and additional support was included from expression levels, CAGE-seq tags and histone modification ChIP-seq assays. Thus, the confidence in the existence and location of the more than 12 thousand confident lncRNA loci is high, even though not all gene boundaries and splice sites might be correct. In fact, the reproducibility of exact lncRNA short-read transcript models between samples was shown to be low in another sheep study (Bush et al., 2018). Related to this, the amount of detected lncRNAs did not reach saturation at any sequencing depth. It has been proposed that, because of stochastic sampling, much higher sequencing depth is needed to reconstruct the vast number of lowly expressed lncRNA transcript models (Bush et al., 2018). It should be mentioned that it is expected that a higher number of the assembled transcripts have independent evidence of expression. On one side, the signal of CAGE-seq scales with expression, similar to RNA-seq so, lowly expressed transcripts are also more weakly represented. On the other, the ChIP-seq dataset used only comprises a single cell type, while the RNA-seq dataset includes several tissue-types.

As observed in other livestock studies (Bush et al., 2018; Kern et al., 2018), the expression levels of lncRNAs were lower than those of PCGs. The lncRNAs already present in the Ensembl annotation were more abundant, were expressed in more samples and were better supported by TSSs and histone modifications, reflecting their misannotation as lncRNA genes when many of them show coding potential. The low expression in bulk RNA-seq samples might be due to their known exceptional cell type, tissue, developmental stage and disease state specific expression (Cabili et al., 2011; Derrien et al., 2012) and even to lowered transcriptional burst frequencies in single-cells (Johnsson et al., 2022). In human and murine T cells and B cells, lncRNAs are expressed in a very cell-specific and dynamic way during differentiation within lineages of the same cell types (Hu et al., 2013; Ranzani et al., 2015; Agirre et al., 2019). In this manner, cell or tissue type specific lncRNAs could be involved in immunological pathways in response to infection and vaccination (de Lima et al., 2019; Du et al., 2020), even if the perceived bulk expression was low.

The biological function of most lncRNAs remains unknown, particularly in non-model organisms. With notable exceptions, few genes can be assigned a putative function by homology with human or mouse lncRNAs. Considering sequence similarity, around 700 novel sheep lncRNA transcripts had orthologues in human, including some functionally characterised lncRNAs, and more than 3,000 in goat or cattle, which are mostly uncharacterised. Because of this, we linked the sheep lncRNAs with potential broad biological functions and pathways by using classical analyses like differential gene expression and co-expression analysis, and alternative methods like differential co-expression network analysis. In the case of the co-expression analyses, following the principle of guilt-by-association (Wolfe et al., 2005), association with the immune response was assigned *via* correlation to a group of co-expressed genes. This approach has been widely used for the functional profiling of lncRNAs by several studies (Walters et al., 2019; de Goede et al., 2021). In addition, one of the datasets included in this study has already been analysed in this way to specifically search for candidate lncRNAs during an helminth infection (Chitneedi et al., 2021).

Regarding the results from the blood cell dataset, with samples from whole blood, PBMCs and other cells like macrophages, all analyses resulted in the identification of genes linked to the innate immune response. Many genes were part of the interferon (IFN)-mediated immune response, which provides a first line of defence against pathogens, from viruses to parasites (Schneider et al., 2014). Upon pathogen detection and IFN stimulation, the transcription of several genes termed as IFN-stimulated genes (ISGs) is activated, which control pathogen infection by targeting pathways necessary for pathogen life cycles. Up to 21 of the most important antiviral ISGs were upregulated in the differential expression analysis, including *ADAR*, *APOBEC3Z1*, *BST2*, *RSAD2*, *MX1*, *MX2*, *IFI6*, *IRF9* or orthologues of the OAS gene family. These genes were part of the iFMDV-associated co-expression module and many were also part of the DCN. The IFN response was mostly driven by the inactivated vaccine (Braun et al., 2018; Jouneau et al., 2020) and the LPS stimulation datasets (Bush et al., 2020), while the helminth infection (Fu et al., 2016; Niedziela et al., 2021) and other smaller datasets (Varela-Martínez et al., 2018; Wang et al., 2019; Guo et al., 2020) produced a different expression profile, as seen in the stimulation-correlated co-expression modules. In the same manner as known ISGs, lncRNAs can also be induced by IFN and have important roles in controlling pathogen infection and resolution of the immune response, or they can regulate the IFN mediated host defence (Meng et al., 2017; Qiu et al., 2018). For instance, in human, *NRIR* is a negative regulator of IFN antiviral response (Kambara et al., 2014) and *IFNG-AS1*, located near the *IFNG* locus, regulates its expression (Petermann et al., 2019). Considering that differentially expressed lncRNAs have been proposed to function as negative or positive regulators in various critical steps of antiviral response (Ouyang et al., 2016), some of the ovine transcripts detected in this study could also be related to those processes.

The fact that the lymph node dataset was dominated by helminth infection experiments (McRae et al., 2016; Chitneedi et al., 2018; Naranjo-Lucena et al., 2021), except for a single bacterial infection experiment (Gossner et al., 2017), greatly marked the type of genes involved in the general analysis. The different analyses revealed important immune-related genes and biological pathways, but there were many other processes involved. Parasite infections produce a different response than viral or bacterial infections and are usually associated with a non-inflammatory Th2-biased response in both parasites present in the datasets: *Teladorsagia circumcincta* and *Fasciola hepatica* (O’Neill et al., 2000; Venturina et al., 2013; Karrow et al., 2014). In a human gene expression meta-analysis with different helminth species, they found upregulated immune regulatory genes while down-regulated genes were mainly involved in metabolic processes, and showed that the response was similar between species and tissues (Zhou et al., 2016). To date, there are very few studies linking lncRNAs to helminth infection in mammals. In sheep, one of the datasets included in this study (Chitneedi et al., 2018) has been analysed for this purpose to specifically search for candidate lncRNAs during *T. circumcincta* infection (Chitneedi et al., 2021). It remains greatly important to identify novel gene candidates for this disease, as it is a source of economic loss and animal welfare deterioration (Karrow et al., 2014).

Integration of several RNA-seq datasets and different bioinformatic analyses allows us to better characterise patterns that could have been overlooked in individual experiments. One of the processes consistently appeared associated with all the analyses in lymph nodes was the response to ER stress, which is an endogenous source of cellular stress that arises in the ER of cells following the accumulation of misfolded proteins during protein synthesis (Todd et al., 2008; Hetz and Papa, 2018). In the immune system, this response is particularly important for resolving secretory stress and survival of highly secretory cells such as immunoglobulin producing plasma cells (Todd et al., 2008), cytokine producing Th2 cells (Pramanik et al., 2018) and other immune cells (Hetz and Papa, 2018). Among the ER stress response-related dysregulated genes, the two most important members of the IRE1a-XBP1 pathway (*ERN1* and *XBP1*) were upregulated in the lymph node samples (Hetz and Papa, 2018) and a co-expression module enriched in ER stress response genes was correlated with both helminth infection and paratuberculosis. This process was enriched in the sets of DEGs in the original analyses of the paratuberculosis dataset (Gossner et al., 2017) and one helminth infection dataset (Fu et al., 2016), but were not further discussed in their respective publications. Furthermore, while belonging to a non-associated co-expression module and not being differentially expressed, the ER localized transcription factor *CREB3* was differentially co-expressed with several other genes. *CREB3* has been implicated in the ER and Golgi stress response and regulation of genes in secretory pathways (Sampieri et al., 2019). LncRNAs have also been linked to proliferation and apoptosis during ER stress (Zhao et al., 2020).

The DCN analyses revealed the involvement of other PCGs and lncRNAs in the ovine immune system activation. Compared to the widely employed co-expression methods, differential co-expression have the advantage of detecting condition-dependent interactions between genes (Savino et al., 2020). For instance, the gain or loss of co-expression between a transcription factor (TF) and its targets can be due to expression changes or post-translational modifications of the TF (Bhuva et al., 2019). Apart from the mentioned *CREB3* transcription factor in lymph nodes, in blood cell samples *IDO1* seemed to be differentially regulated. *IDO1* is a rate-limiting metabolic enzyme that converts tryptophan into downstream kynurenines, which have immunosuppressive roles, and is known to be interferon-inducible (Zhai et al., 2018). Similarly to the general differential expression analysis in this study, the original analysis of LPS effect on macrophages did not find an induction of *IDO1* expression, even if it was expected (Bush et al., 2020). Interestingly, we found that *IDO1* was part of a co-expression module associated with immune stimulation and enriched in ISGs, and was differentially correlated with several genes. In stimulated samples its expression was independent from other genes, but upon immune stimulation it gained correlations with genes like the ISG *DDX58*. All in all, the constructed DCNs revealed several lncRNAs with stimulation-dependent associations that could have immune regulatory roles, and this approach could be useful to find novel gene candidates in each pathogen infection or vaccine component.

## Conclusion

Multiple processes are involved in the immune response to infection and vaccination and lncRNAs might play different roles in these processes. The goals of this work were 1) to detect unannotated ovine lncRNAs from publicly available RNA sequencing datasets from immune tissues and then 2) define a lncRNA gene expression signature of the general immune activation. Poor sequence conservation and low expression, general features found in other mammal studies, were also features of ovine lncRNAs. Adding support from CAGE sequencing and histone modifications, we obtained a shortlist of more than 12 thousand unannotated high-confidence ovine lncRNAs. The functional analyses performed with immune-stimulated samples revealed hundreds of known and novel lncRNAs with specific expression patterns during an infection or vaccination. These genes make up a prioritized set of potential candidates for deeper experimental analyses. Taken together, these results should help completing the sheep non-coding RNA gene catalogue, and most importantly, they give evidence of immune state-specific lncRNA expression patterns in a livestock species.

## Data Availability

Publicly available datasets were analyzed in this study. This data can be found here: The RNA-seq datasets analysed in this work were obtained from the NCBI SRA repository under the following project accessions: PRJEB26387, PRJNA454435, PRJNA559411, PRJNA291172, PRJNA433706, PRJNA268183, PRJEB33476, PRJEB45790, PRJEB44063, PRJEB15872, PRJNA631066, PRJNA528905, PRJNA485657, PRJNA362606 and PRJEB19199. FAANG CAGE-seq and ChIP-seq datasets were obtained from European Nucleotide Archive (ENA) project accessions PRJEB34864 and PRJEB40528. Annotations and expression quantification of unannotated lncRNAs can be found at https://doi.org/10.5281/zenodo.6802781 (Bilbao-Arribas and Jugo, 2022). The workflow and custom scripts used in this manuscript are available at https://github.com/bilbaom/immune-lncrnas-sheep.
