# Supplementary material for: Transcriptomic meta-analysis reveals unannotated long non-coding RNAs related to the immune response in sheep
Source: Front Genet. 2022 Nov 22;13:1067350. doi: 10.3389/fgene.2022.1067350 (PMC9725098; doi:10.3389/fgene.2022.1067350)
Supplement: Supplementary file 4 [file DataSheet1.PDF]

Supplementary Material

1 Supplementary Figures

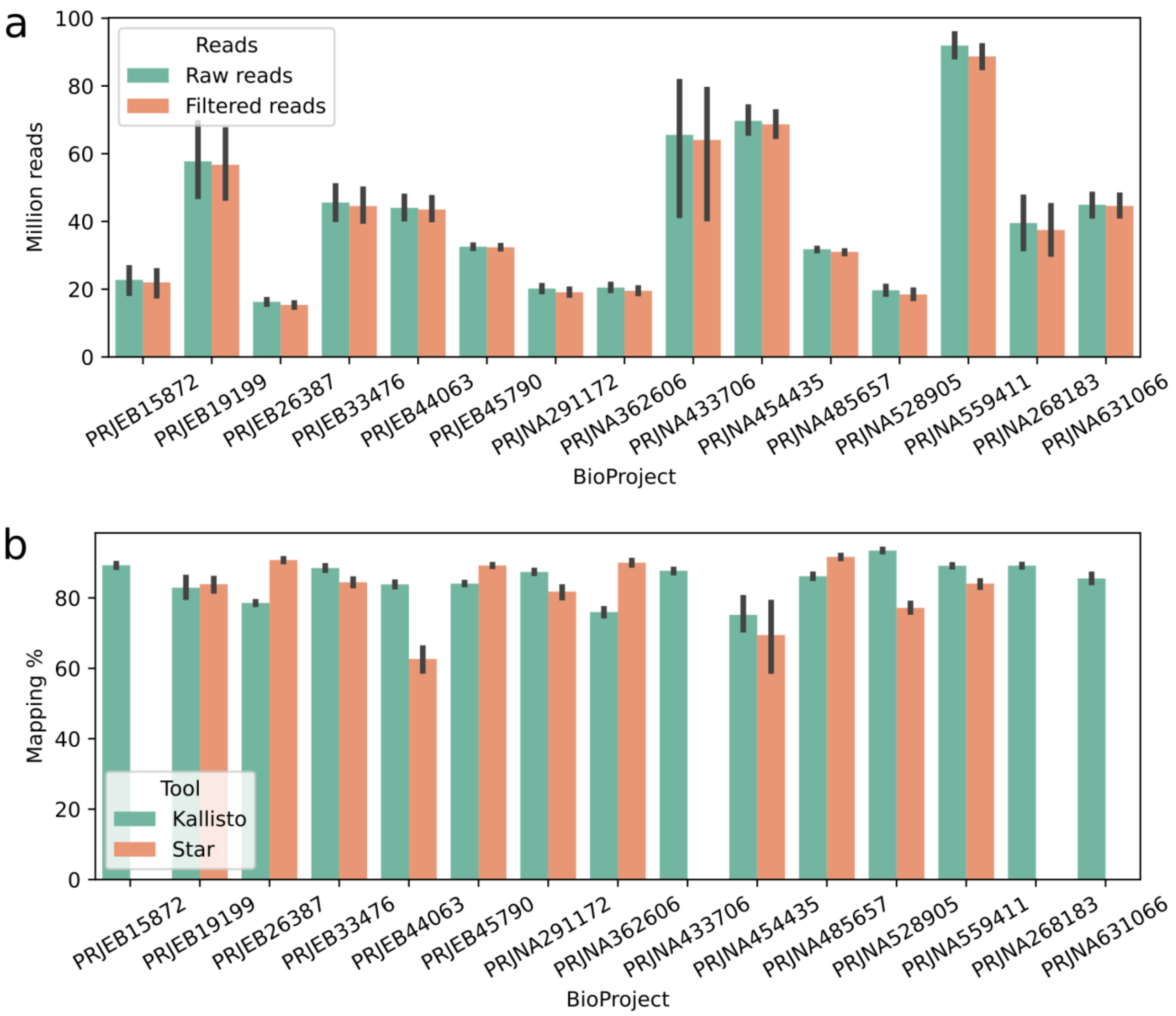

Supplementary Figure 1. Summary statistics of the samples included in the study. (A) Average number of reads in each dataset before and after quality filtering and read adapter removal. (B) Average mapping rate to the genome (STAR) and average pseudo-alignment rate (Kallisto) to the full unfiltered transcriptome assembled with Stringtie. Unstranded samples were not used in the genome mapping for lncRNA identification.

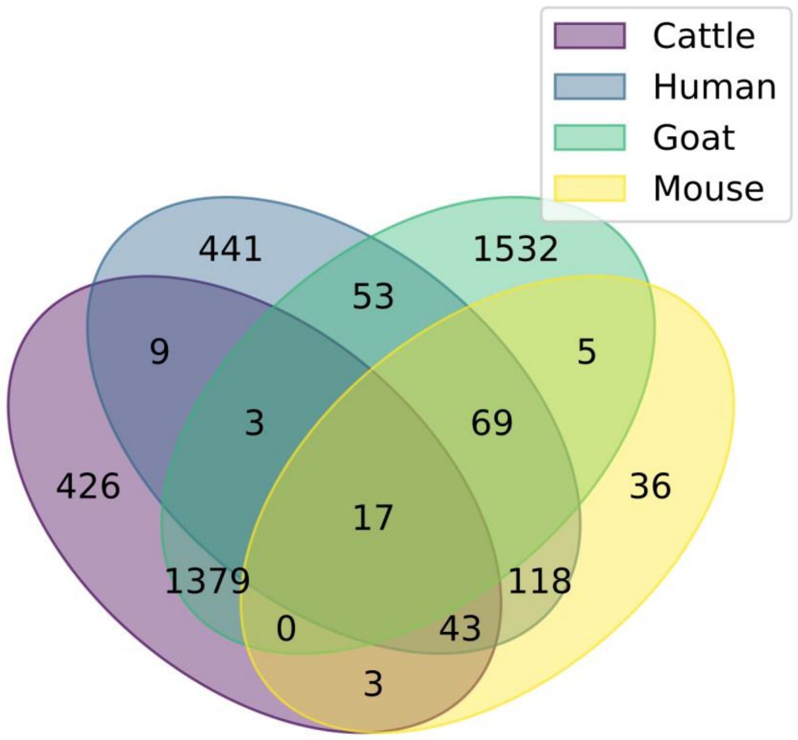

Supplementary Figure 2. Summary of sequence conservation analysis. Number of sheep lncRNA transcripts with significant sequence similarity with annotated lncRNAs in other mammal species.

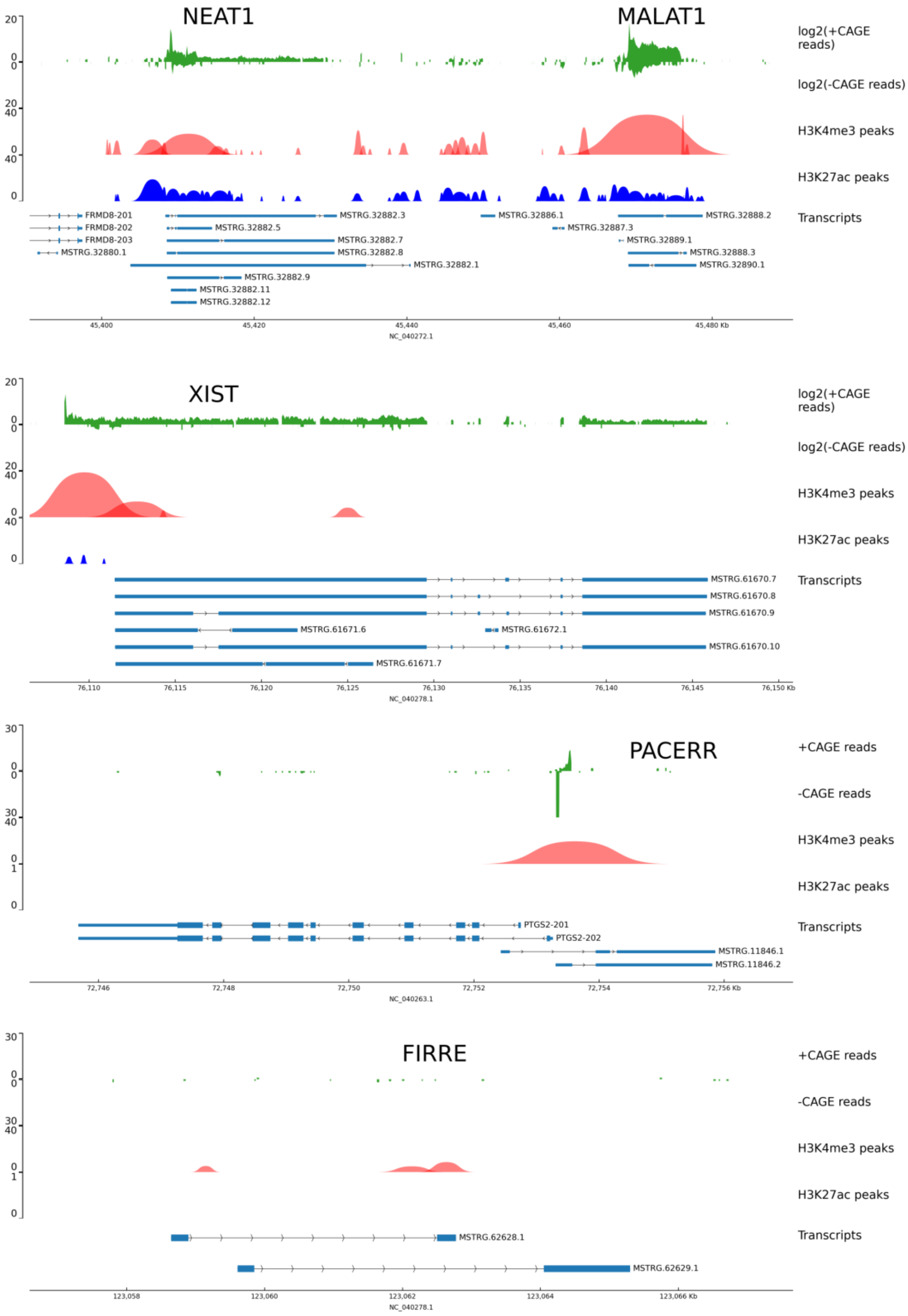

Supplementary Figure 3. Examples of conserved lncRNAs. Genomic context of selected conserved lncRNAs between sheep and human is depicted, including CAGE-seq read mapping, predicted CHIP-seq peaks and transcripts models.

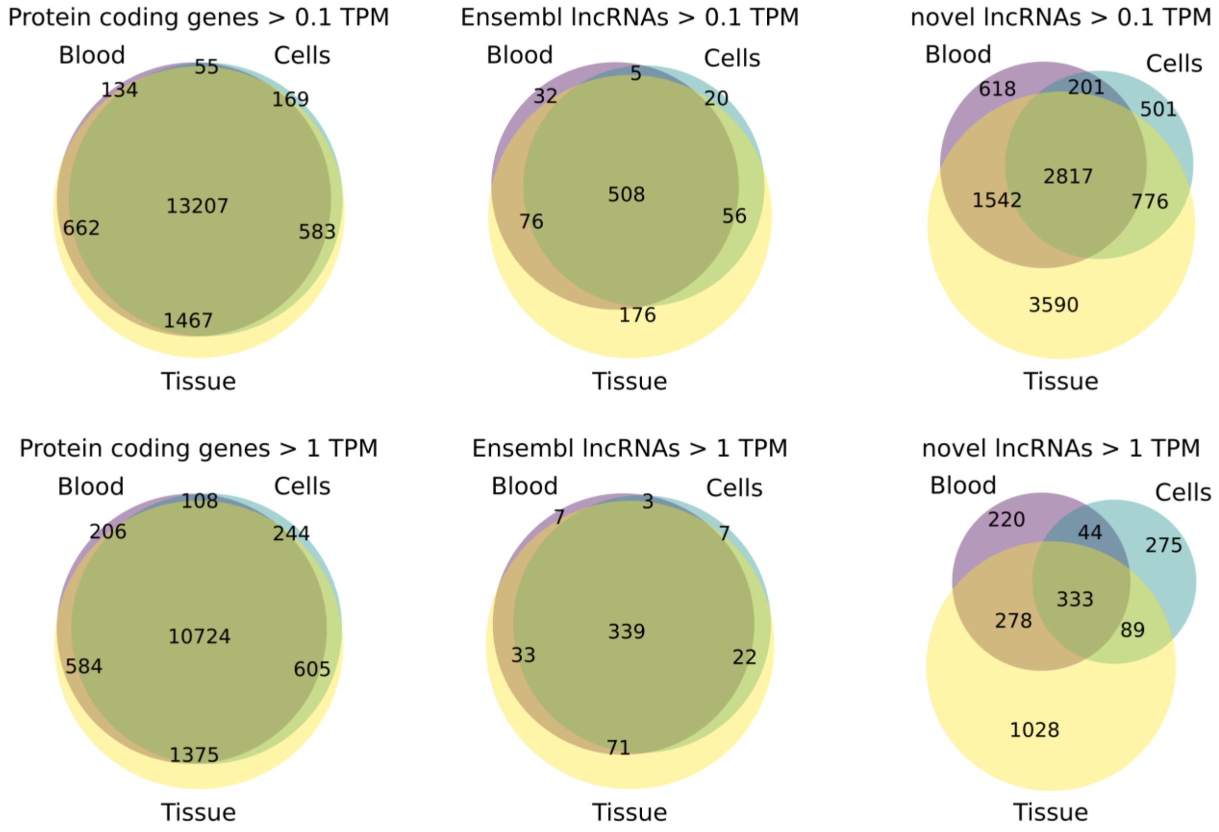

Supplementary Figure 4. Expression Venn diagrams. Venn diagrams comparing the expression of PCGs, annotated lncRNAs and novel lncRNAs in each tissue group.

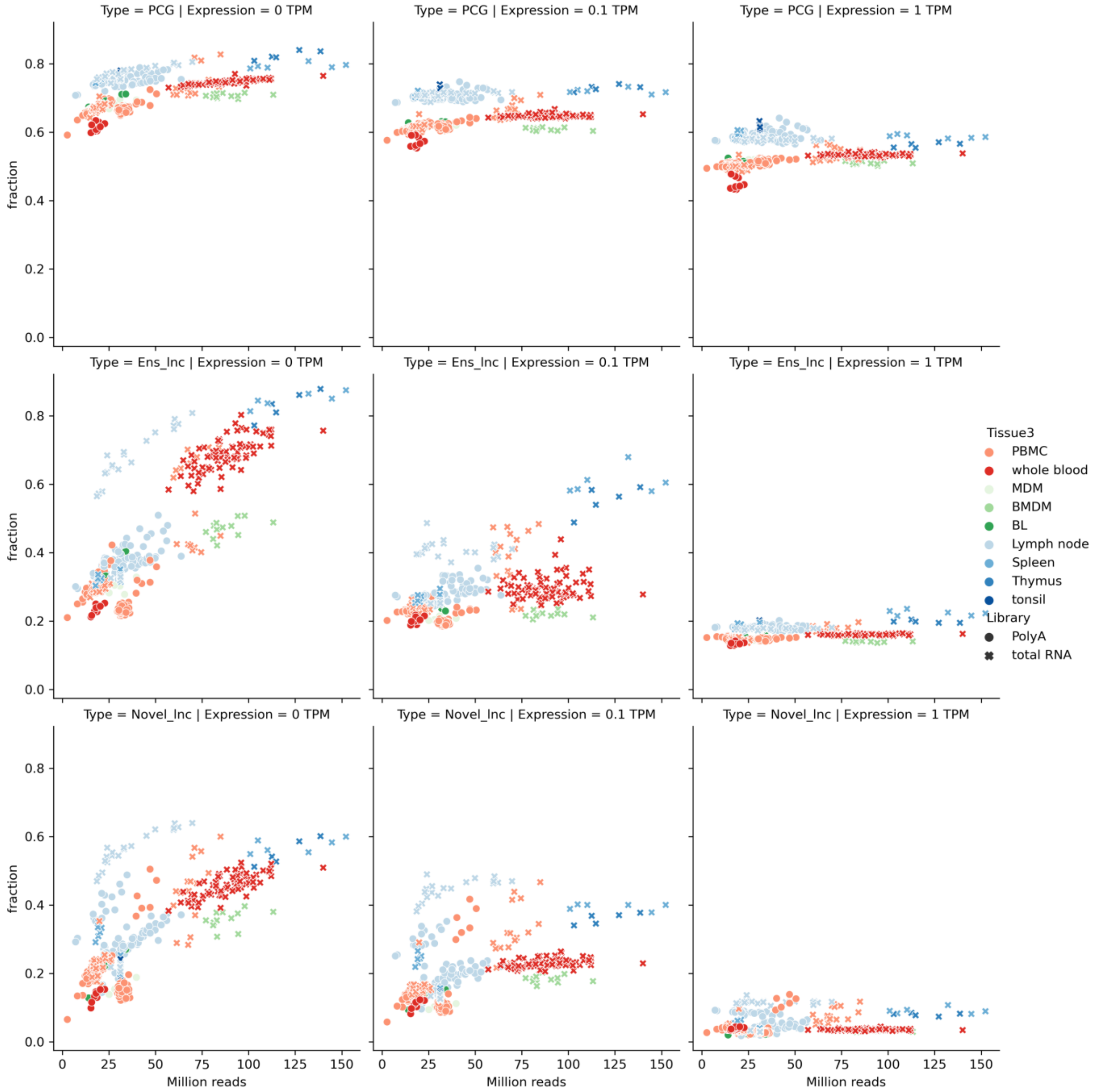

Supplementary Figure 5. Saturation of gene detection. Number of expressed PCGs and lncRNAs in each sample as a fraction of all genes annotated from each type compared to sequencing depth of the samples.

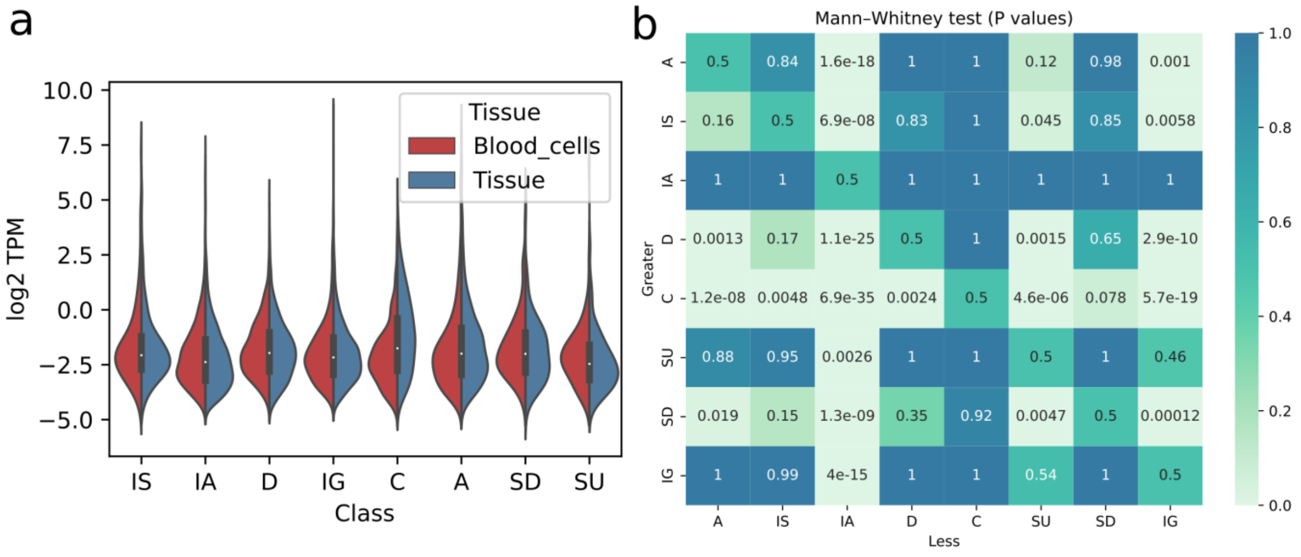

Supplementary Figure 6. Expression of lncRNA classes. (A) Expression of each class of unannotated lncRNAs in blood and cell samples and in lymphoid tissues. LncRNAs expressed above 0.1 TPM in at least one fifth of the samples in each tissue group were used. (B) One-sided Mann-Whitney U tests between the expression levels of each class of unannotated lncRNAs. LncRNAs expressed above 0.1 TPM in at least one tenth of the samples were used. Class codes: intronic sense (IS), intronic antisense (IA), divergent (D), intergenic (IG), convergent (C), antisense (A), sense downstream (SD) and sense upstream (SU).

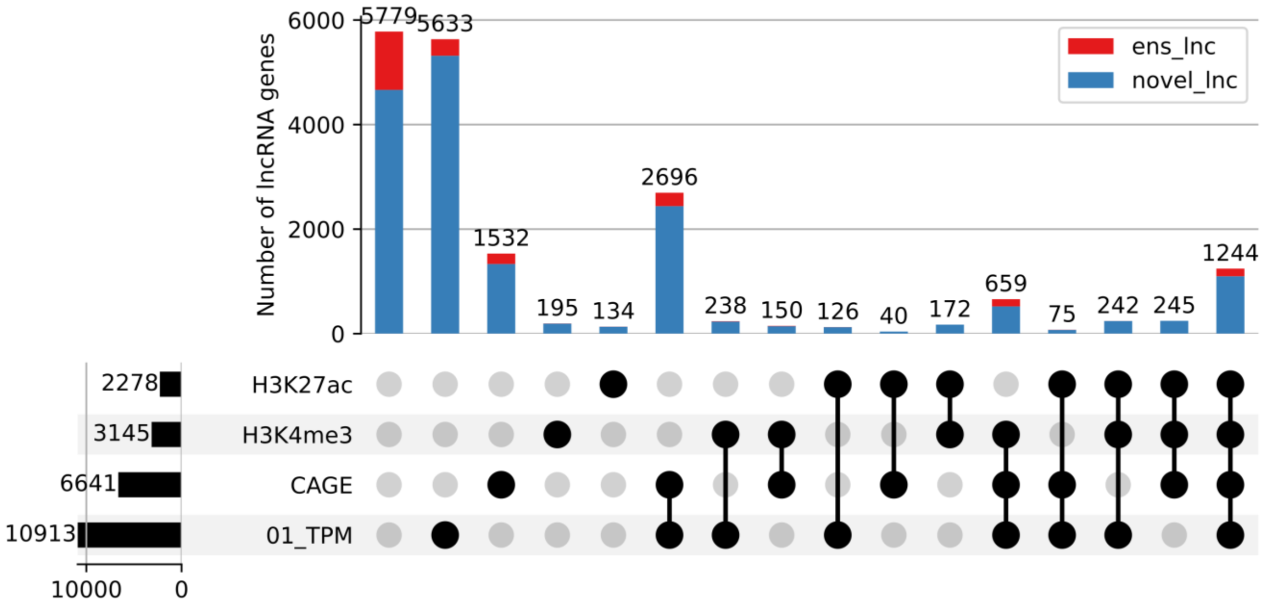

Supplementary Figure 7. Intersections of support from ChIP-seq histone modifications and CAGE-seq peaks in all annotated and novel lncRNAs.

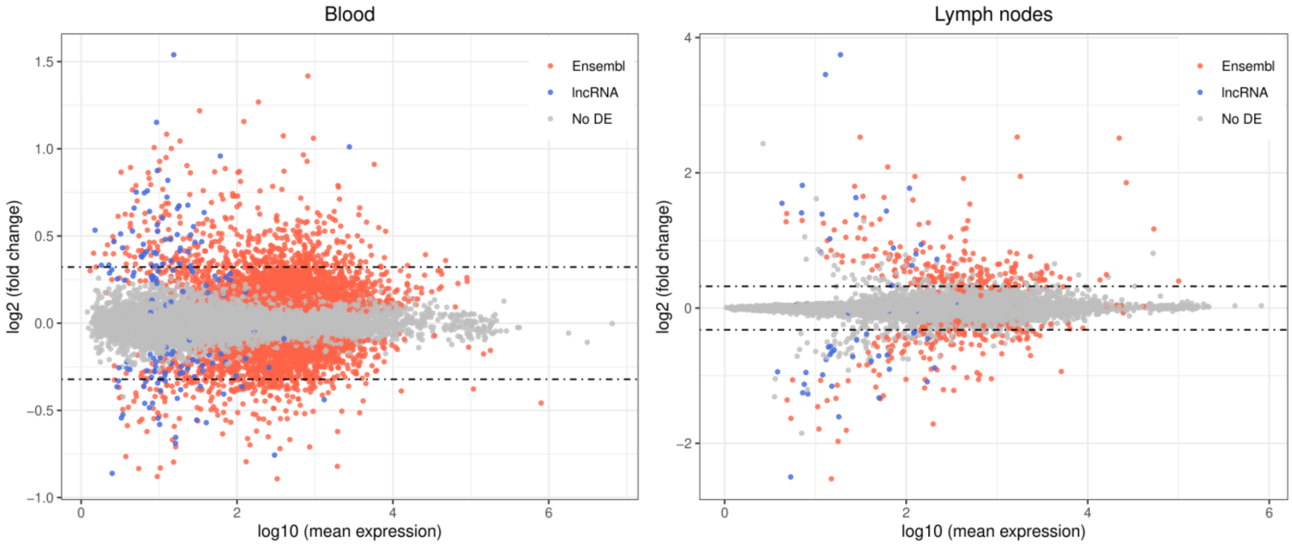

Supplementary Figure 8. MA plots of the differential-expression results in blood cells and lymph nodes.

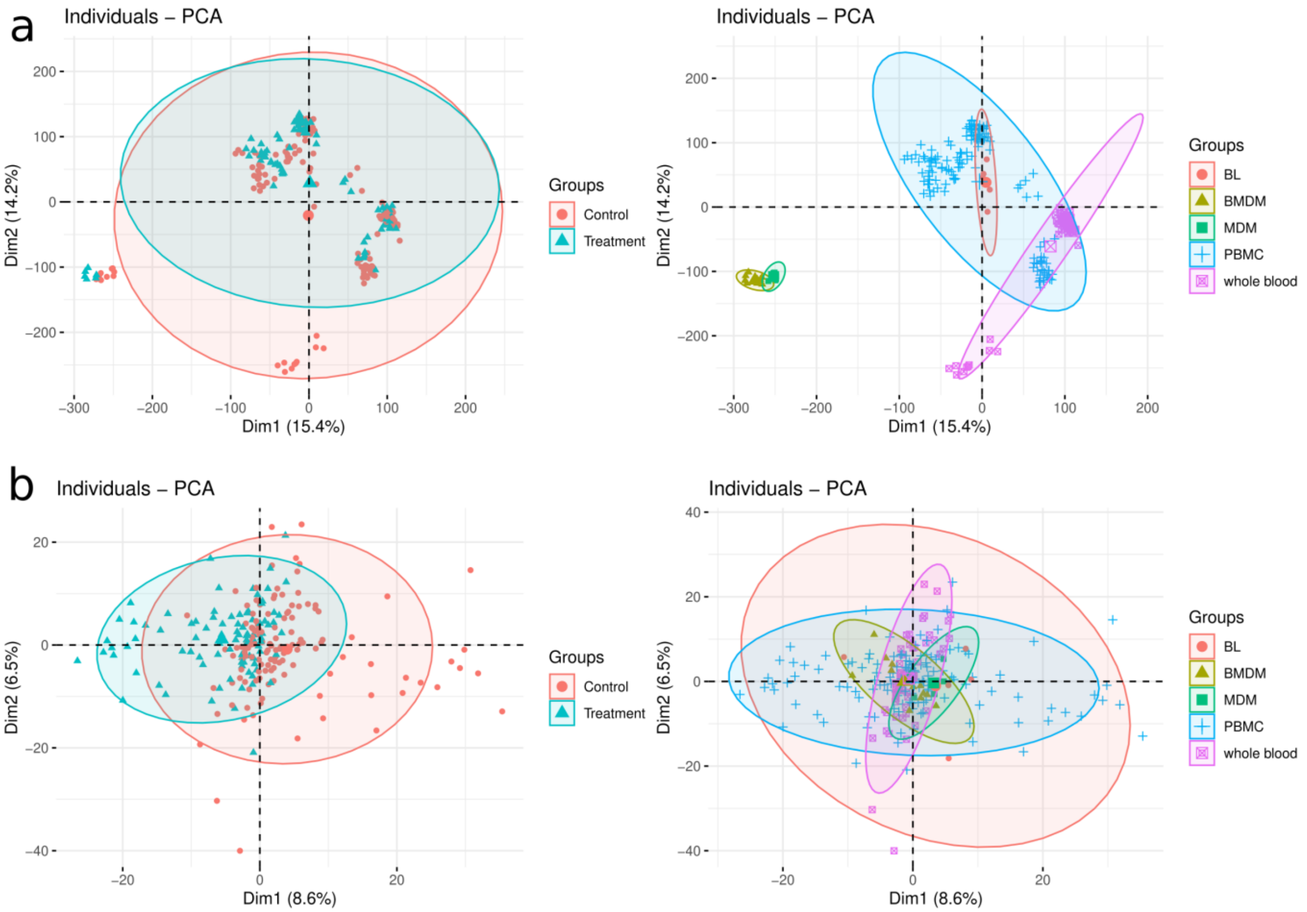

Supplementary Figure 9. PCA plots of the blood cell samples used for coexpression analysis, coloured by treatment group and tissue. (A) Samples before any correction. (B) Samples after correction for covariates.

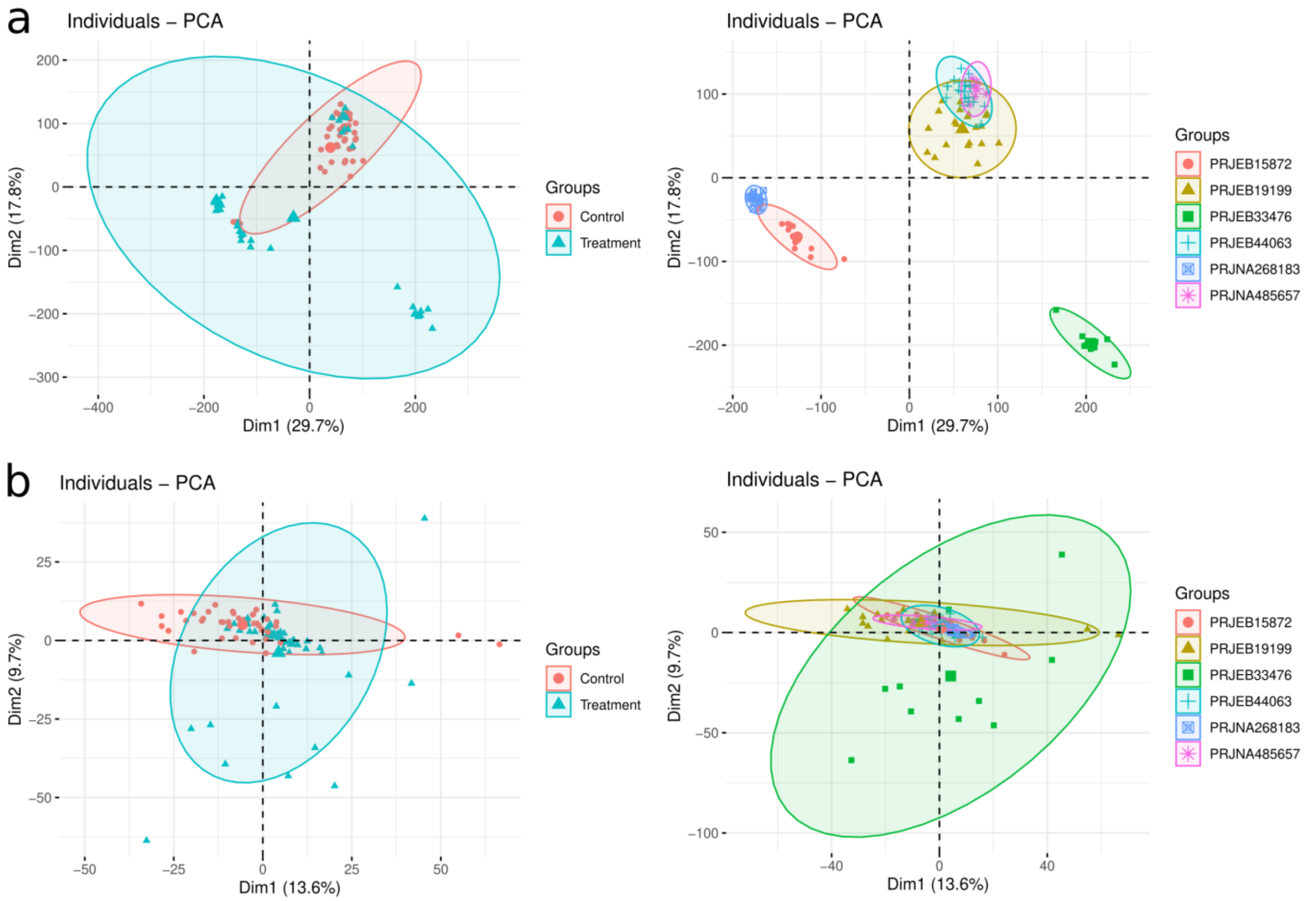

Supplementary Figure 10. PCA plots of the lymph node samples used for coexpression analysis, coloured by treatment group and project accession. (A) Samples before any correction. (B) Samples after correction for covariates.

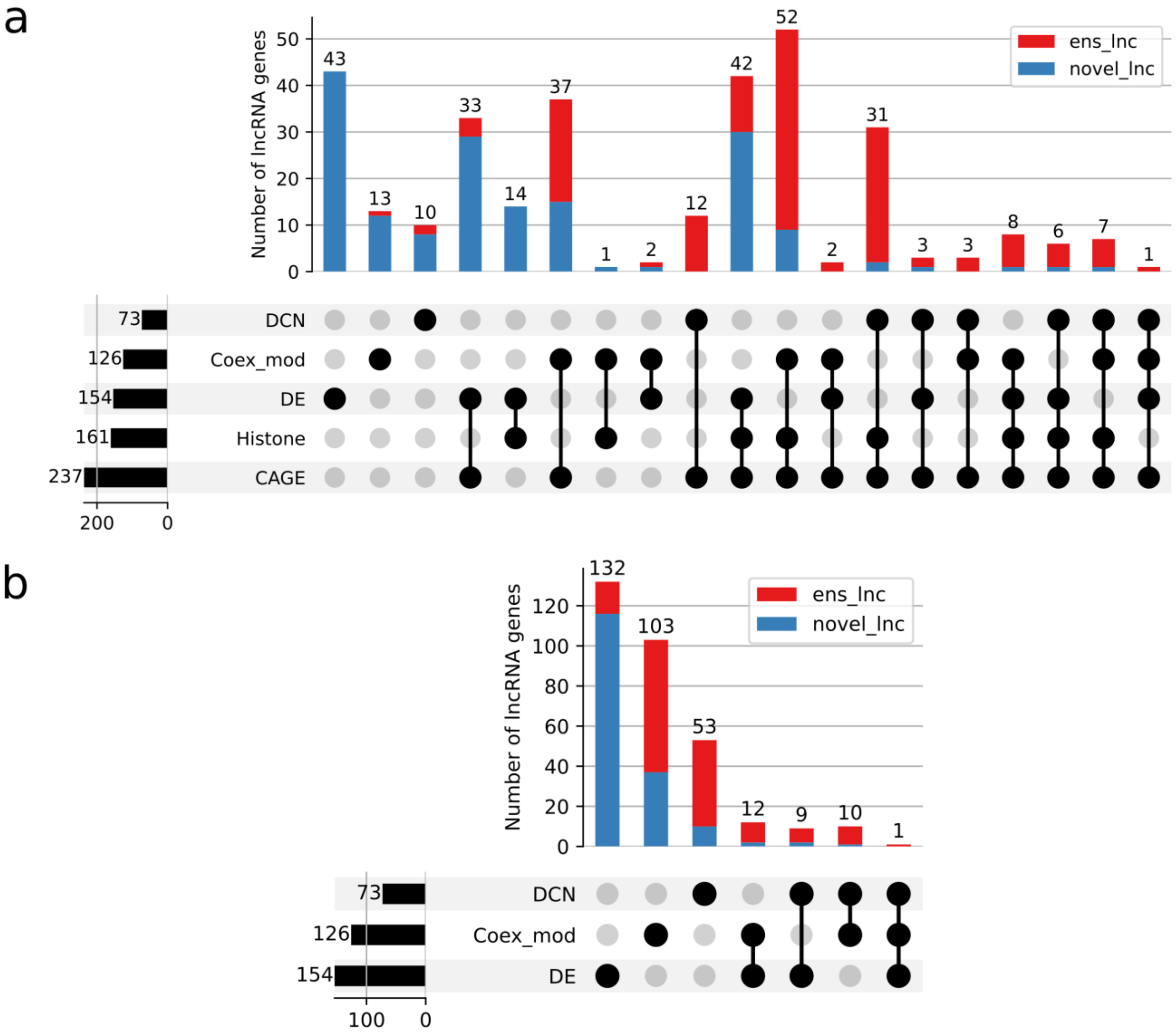

Supplementary Figure 11. Upset plots of the integration of lncRNA features and the functional analyses. (A) Intersections of statistically significant genes from differential expression analysis (DE), immune-enriched modules (Coex_mod) and differential co-expression analysis (DCN) with CAGE peaks and histone modifications. Annotated and novel lncRNAs statistically significant for at least one functional analysis are depicted. (B) Intersections of statistically significant genes from differential expression analysis (DE), immune-enriched modules (Coex_mod) and differential co-expression analysis (DCN) in annotated and novel lncRNAs.
